# Efficiency and Power as a Function of Sequence Coverage, SNP Array Density, and Imputation

**DOI:** 10.1371/journal.pcbi.1002604

**Published:** 2012-07-12

**Authors:** Jason Flannick, Joshua M. Korn, Pierre Fontanillas, George B. Grant, Eric Banks, Mark A. Depristo, David Altshuler

**Affiliations:** 1 Broad Institute of Harvard and MIT, Cambridge, Massachusetts, United States of America; 2 Department of Molecular Biology and Diabetes Unit, Massachusetts General Hospital, Boston, Massachusetts, United States of America; 3 Harvard-MIT Division of Health Sciences and Technology, Cambridge, Massachusetts, United States of America; 4 Graduate Program in Biophysics, Harvard University, Cambridge, Massachusetts, United States of America; 5 Department of Genetics and Medicine, Harvard Medical School, Boston, Massachusetts, United States of America; EMBL, Germany

## Abstract

High coverage whole genome sequencing provides near complete information about genetic variation. However, other technologies can be more efficient in some settings by (a) reducing redundant coverage within samples and (b) exploiting patterns of genetic variation across samples. To characterize as many samples as possible, many genetic studies therefore employ lower coverage sequencing or SNP array genotyping coupled to statistical imputation. To compare these approaches individually and in conjunction, we developed a statistical framework to estimate genotypes jointly from sequence reads, array intensities, and imputation. In European samples, we find similar sensitivity (89%) and specificity (99.6%) from imputation with either 1× sequencing or 1 M SNP arrays. Sensitivity is increased, particularly for low-frequency polymorphisms (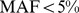), when low coverage sequence reads are added to dense genome-wide SNP arrays — the converse, however, is not true. At sites where sequence reads and array intensities produce different sample genotypes, joint analysis reduces genotype errors and identifies novel error modes. Our joint framework informs the use of next-generation sequencing in genome wide association studies and supports development of improved methods for genotype calling.

## Author Summary

In this work we address a series of questions prompted by the rise of next-generation sequencing as a data collection strategy for genetic studies. How does low coverage sequencing compare to traditional microarray based genotyping? Do studies increase sensitivity by collecting both sequencing and array data? What can we learn about technology error modes based on analysis of SNPs for which sequence and array data disagree? To answer these questions, we developed a statistical framework to estimate genotypes from sequence reads, array intensities, and imputation. Through experiments with intensity and read data from the Hapmap and 1000 Genomes (1000 G) Projects, we show that 1 M SNP arrays used for genome wide association studies perform similarly to 1× sequencing. We find that adding low coverage sequence reads to dense array data significantly increases rare variant sensitivity, but adding dense array data to low coverage sequencing has only a small impact. Finally, we describe an improved SNP calling algorithm used in the 1000 G project, inspired by a novel next-generation sequencing error mode identified through analysis of disputed SNPs. These results inform the use of next-generation sequencing in genetic studies and model an approach to further improve genotype calling methods.

## Introduction

High coverage whole genome sequencing gives maximum information about individual-level genetic variation for use in disease gene mapping [1]–[6] or population genetics [7]–[12]. However, due to cost limitations, most population-based studies have estimated sample genome sequences through incomplete data collection with SNP arrays [13], [14], low coverage whole genome sequencing [15], [16], or high coverage targeted sequencing [17]–[20]. To increase power, such studies often combine sample genotypes with well-characterized public reference panels [9], [10], [21] and use statistical imputation [22], [23] to predict missing or unassayed genotypes. It is as yet unclear how much power is lost, and how much efficiency is gained, by these incomplete data collection strategies.

The literature on analytical methods for low coverage sequencing and SNP arrays has until now been largely non-overlapping [14], [24]–[29]. Past studies have investigated the power and efficiency of each technology separately: how well subsets of common variants [30]–[33] or commercially available SNP arrays [34] “tag” common variants in the genome and empower genome-wide association studies [35], [36], the power of low coverage sequencing relative to high coverage sequencing [37], differences in performance of whole-genome sequencing technologies [38], and the performance of imputation [39] and its impact on power [25], [40], [41]. As most investigators must decide among these technologies, we sought to compare their power and efficiency within a unified analytical framework. Moreover, as genome wide association studies (GWAS) have been performed on hundreds of thousands of valuable clinical samples, and as investigators now must choose whether to collect additional data on these samples with next-generation sequencing, we sought to evaluate the benefit of combined sequence and SNP array data collection strategies. Finally, we sought to exploit the existence of sequencing and array data in a common set of samples to identify error modes and thereby improve genotype calling algorithms.

Specifically, we asked four questions: (1) as a baseline, how does power (sensitivity and specificity of genotype calls) vary based on sequence read depth and SNP array density, with and without imputation; (2) do combinations of different technologies have improved performance relative to each data type alone; (3) when sequencing and arrays disagree, is it possible to identify the incorrect technology; and (4) can we learn and ultimately resolve new error modes based on these disagreements?

## Results

**Figure 1:**
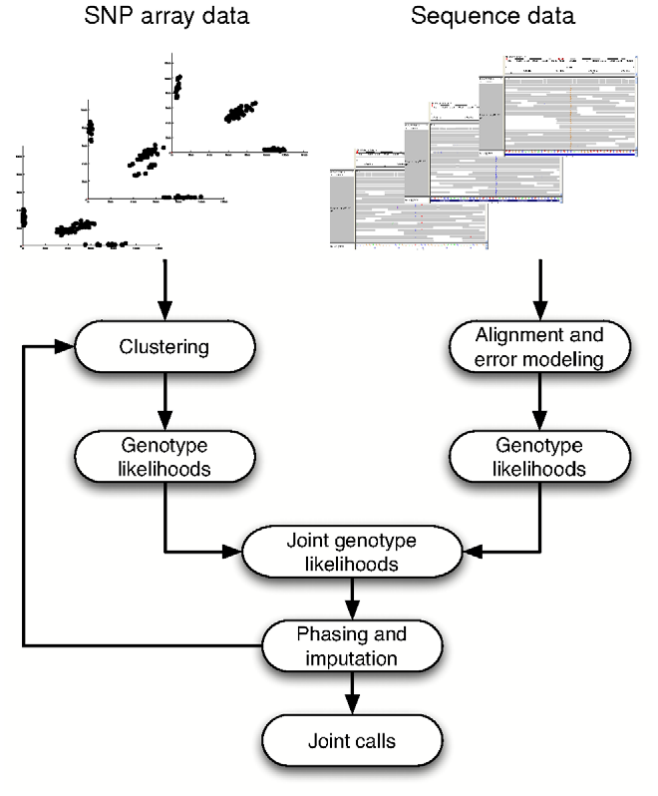
A statistical framework for joint genotype calls. We developed a statistical framework to jointly estimate sample genotypes from array intensities, sequence reads, and haplotype phasing. The framework first estimates genotype likelihoods independently for each SNP from array and sequence data, given initial parameters for genotype cluster locations and sequence read error rates. It then multiplies the likelihoods for each SNP to obtain joint likelihoods, inputs these to haplotype phasing and imputation, and then uses the output likelihoods to re-cluster intensities for all SNPs. The process is iterated, and upon termination genotype likelihoods are converted to posterior genotype probabilities. The framework can estimate genotypes given only sequence data or array data as well as with or without imputation — many of these special cases are similar in principle to previously described genotyping algorithms (Text S1).

We first developed a statistical framework that can jointly estimate genotypes from array intensities, sequence reads, and imputation (Figure 1). The framework first estimates genotype likelihoods for each SNP independently from array intensities and/or sequence reads and then combines these likelihoods to produce joint likelihoods. The array genotype cluster locations and the joint likelihoods are iteratively re-estimated using an Expectation-Maximization algorithm with haplotype phasing and imputation (Materials and Methods). In this manner, sequence reads, intensities from SNP arrays, and haplotype phasing jointly inform the genotype likelihood for each site in each individual (Figure S1). We developed an implementation of this framework that uses published methods for SNP array clustering [14], sequence SNP calling [27], and imputation [42] to call sample genotypes (Figure S2); source code is available from the authors upon request.

This framework produces an integrated estimate of the sequence variation in each individual, enabling comparisons among technologies. We used it to perform a series of experiments, modeling different array or sequence data collection strategies. Using raw array intensities and sequence reads from the Hapmap [9], [21] and 1000 Genomes (1000 G) projects [10], we evaluated six SNP arrays (five genome-wide with 100 k to 2.5 M SNPs, one the custom Metabochip), four levels of whole-genome Illumina sequence coverage (.5× - 4× reads per base), and all combinations thereof. In each analysis we called genotypes on chromosome 20 for 382 unrelated European samples from phase 1 of the 1000 G Project. In our primary analysis, we only called genotypes with posterior probability above 90%; the remainder were considered missing (no-calls). In addition, the current experiments evaluate SNPs only (i.e. they do not consider insertions, deletions, or structural variation) and are limited to autosomal chromosomes. Full details of our experimental setup are given in Materials and Methods; analysis of the impact of different parameters, such as the number and ethnicity of samples genotyped, is provided in Text S1.

To evaluate performance (relative to high coverage whole genome sequencing), we compared the resulting genotype calls for a single test sample to “gold-standard” genotypes, published from multiple high coverage sequencing technologies by the 1000 Project Pilot, for the same sample. We focused our metrics only on non-reference alleles. To measure sensitivity (how well a strategy identifies all true non-reference alleles) we defined 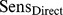 (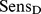) and 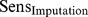 (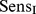) as the fraction of non-reference gold-standard genotypes with identical called genotypes (without and with imputation respectively). Conversely, to measure specificity (how well a strategy identifies only true non-reference alleles), we defined 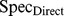 (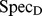) and 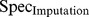 (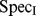) as the fraction of non-reference called genotypes with identical gold-standard genotypes (without and with imputation). Further details of our experimental procedure are given in Materials and Methods and Figure S3.

### Evaluation of sensitivity and specificity

**Figure 2:**
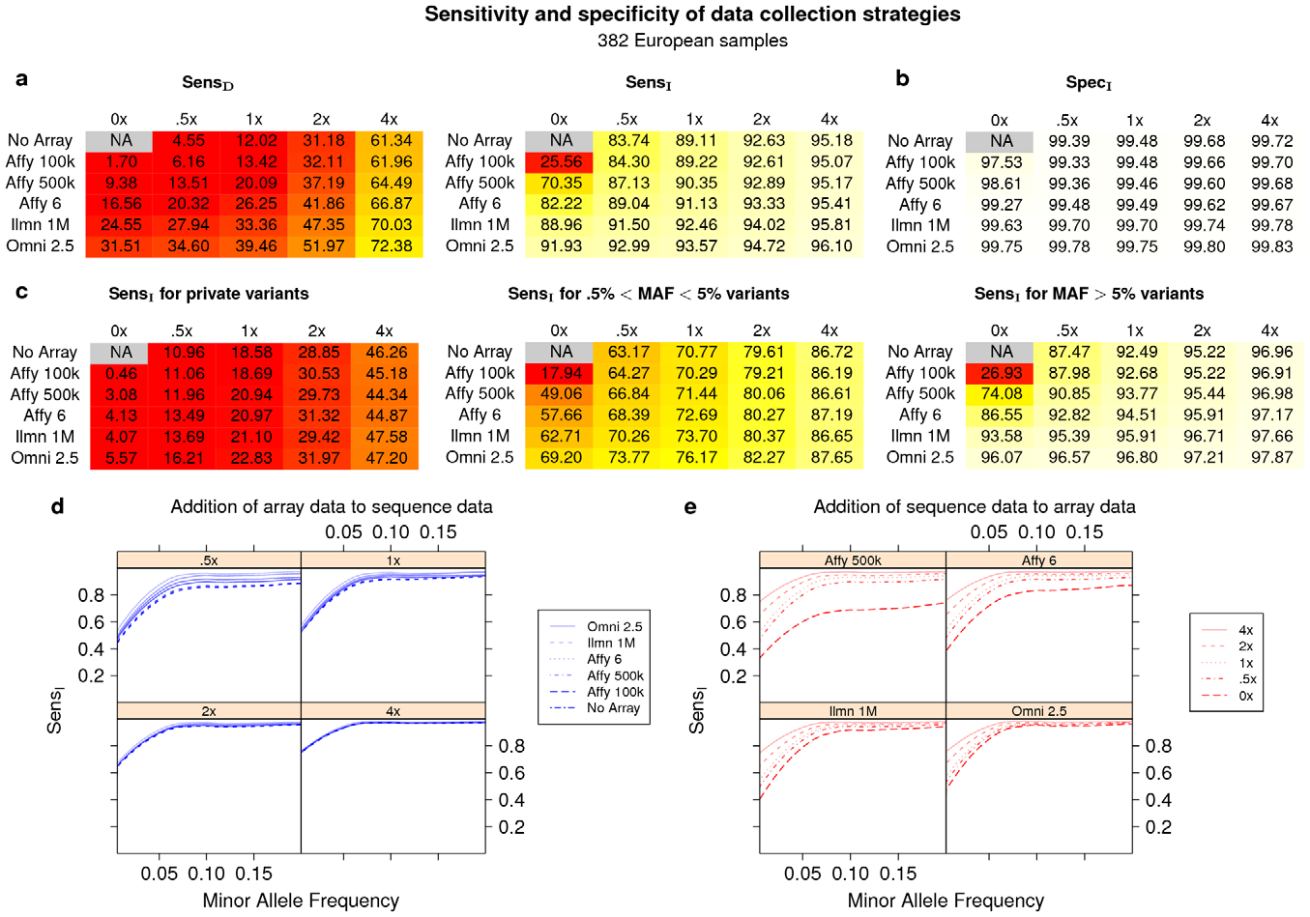
Sensitivity and specificity of data collection strategies. For different combinations of array and sequence data, we produced joint genotype calls on chromosome 20 for 382 European samples from the 1000G project. For a single test sample, we obtained “gold-standard” genotypes from high coverage multi-technology sequencing published by the 1000G project. We then measured non-reference site sensitivity and specificity with imputation (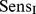, 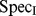) and without (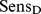, 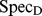). (**a**) 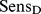 (left) and 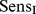 (right) of calls from five array densities and four sequence coverages. The first row of each table contains results for strategies with only sequence data, and the first column contains results for strategies with only array data. A common color scheme is used across all tables, with white corresponding to 100%, red corresponding to 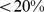, and yellow corresponding to 80%. (**b**) 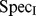 of calls; 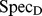 is given in Figure S9. (**c**) 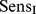 for three variant frequency ranges, with frequency estimated from the non-test samples. Private variants have frequency 0% in the non-test samples. (**d**) 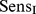 for four sequence coverages, with separate lines that correspond to joint calls made with each SNP array. (**e**) 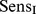 for four array densities, with separate lines that correspond to joint calls made with each sequence coverage. No Array: from sequence data alone; 0×: from array data alone; .5×-4×: mean number of sequence reads per genomic position; array abbreviations are defined in Materials and Methods.

We first assessed the sensitivity of each technology. Figure 2a and Figures S4, S5, S6, S7, S8, S9 show that values of 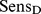 vary widely, increasing roughly linearly with array density from 1.7% to 31.51% (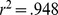) and roughly linearly with sequence coverage from 4.55% to 61.34% (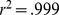). However, 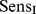 values are dramatically higher than, and increase less steeply with, density and coverage (26–92% for arrays, 84%–95% for low coverage sequencing). Strikingly, even low depths of sequence coverage (0.5×) provide high values for 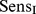 (83.33%). These results reflect that most genetic variation in any given European individual is common and redundant due to LD, and such variation is extracted effectively using imputation with access to data from the 1000 G Project.

Based on the clear value and wide availability of methods and data for statistical imputation, from this point forward we discuss only calls made using imputation.

We next assessed the specificity of each technology. Using a 90% posterior probability no-call threshold, 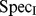 values are above 99% for almost all assays. Completely eliminating the no-call threshold increases 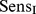 substantially but at the cost of 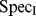 decreased to 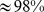 (Figures S10, S11). While overall specificity remains high, the specificity of marginal calls added due to the reduced threshold is lower (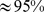). Based on individual aims, investigators must thus choose posterior probability thresholds to trade off sensitivity for specificity.

Our analysis allowed direct comparison of low coverage sequencing and SNP arrays within a common framework (Figure 2ab). For example, we find that 0.5× sequence coverage is more sensitive than 500 k SNP arrays (84% vs. 70% 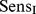), that 1× sequencing is comparable to a 1 M SNP array (89% 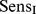), and that 2× sequencing is similar to 2.5 M SNP arrays (93% vs. 92% 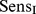). Although all technologies have higher sensitivity for variants with non-reference homozygous genotypes than at variants with heterozygous genotypes, the relative sensitivities of the technologies are roughly the same for both variant classes (Figure S12). Thus, sequencing to one-half or one-quarter of coverage previously proposed for complex trait association studies [37] gives comparable sensitivity to SNP arrays used successfully for GWAS.

Notably, performance is different for coding variants — which constitute 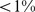 of variant sites in our test sample but are a high priority for some genetic studies [5]. In this setting, 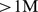 SNP arrays and 4× sequencing have significantly higher sensitivities for coding variants relative to noncoding variants (Figure S13). In contrast, 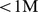 arrays arrays or 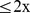 sequencing have slightly lower sensitivities for coding variants relative to noncoding variants. As a result, for coding variants alone, 2× sequencing (in contrast to 1× sequencing) is comparable to a 1 M SNP array.

We observe that different methods, even those with similar sensitivities across all variants, perform differently across the frequency spectrum (Figure 2c, Figure S14). In particular, for low frequency polymorphisms (MAF .5-5%), low coverage sequencing far outperforms current SNP arrays after imputation with 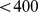 European samples. For example, 1× coverage provides similar 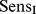 (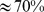) and 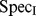 (99.5%) to the densest current arrays (2.5 M SNPs), and 4× coverage far outperforms both (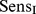 86%, 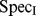 99.5%).

Based on the different sensitivities of SNP arrays and sequencing across the frequency spectrum, we asked if calls made jointly from the two technologies might have higher sensitivity and specificity than calls from either technology alone. We find that, regardless of the array density or sequence coverage, joint calls uniformly have higher 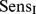 and 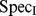 than calls made using only a single technology. However, for any allele frequency, the effect of added array data is exceedingly small once sequence coverage is 2× or greater (Figure 2d). In contrast, addition of sequencing data to first generation (500 k) arrays has substantial benefits across the entire allele frequency spectrum, and even addition to the densest current arrays (2.5 M) substantially increases performance at sites with minor allele frequency 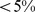 (Figure 2d).

**Figure 3:**
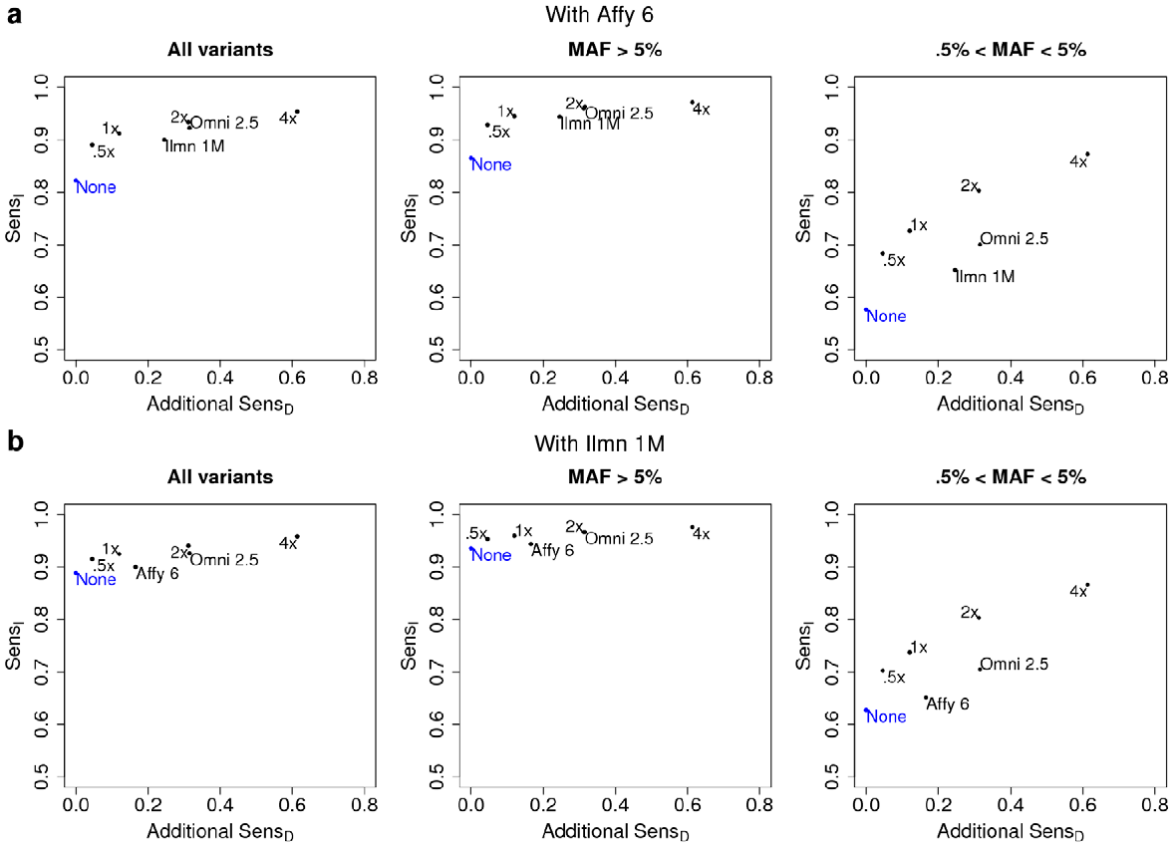
Data collection strategies for studies with prior array data. For the (**a**) Affy 6 and (**b**) Ilmn 1 M arrays, we produced joint calls after addition of each sequence coverage or 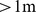 array; joint calls with multiple arrays include combined data from both arrays. The y-axis shows 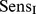, while the x-axis shows 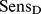 of the additional data collected. 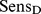 is a measure of the genotyping investment intrinsic to a technology that serves as a proxy for cost. The blue point (None) shows 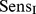 if no additional data is collected; the other points are labeled with the additional data collected. Labels are defined in 2.

These results are particularly relevant in light of the hundreds of thousands of samples previously characterized using first generation SNP arrays for GWAS [43]. As compared to additional higher density genome-wide array-based genotyping, low coverage whole genome sequencing appears to result in greater improvement in sensitivity and specificity per individual (post imputation; Figure 3, Figure S15). The performance increase is greatest for low frequency polymorphisms, a high priority target for many post-GWAS experiments [43]–[47]. We note, however, that our metrics do not measure how many samples have genotype calls at each variant site — arrays genotype each sample at the same set of sites, while low coverage sequencing may genotype each sample at a slightly different set of sites. For some studies, the benefit of having a uniform set of sites genotyped may outweigh the higher per-sample sensitivity of low coverage sequencing.

### Analysis of SNP array and sequencing error modes

In addition to increased sensitivity, joint analysis of low coverage sequencing and array data in the same samples can identify technology-specific error modes at the small number of sites with disputed genotypes. Such analysis can reduce genotype errors in these samples, through resolution of these disputes, but more importantly can spur development of improved genotyping algorithms for each technology.

**Figure 4:**
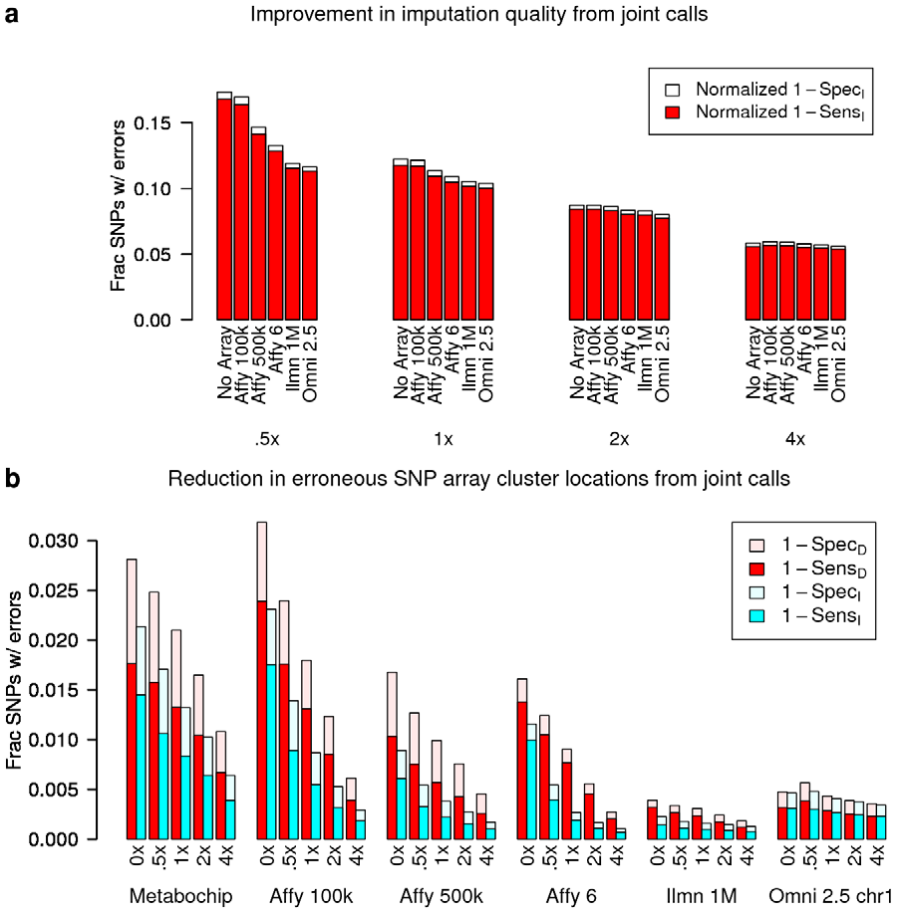
Reduction in errors from joint genotype calls. (**a**) To assess the improvement in imputation quality afforded by joint genotype calls with a SNP array (relative to calls based on sequence data alone), we measured sensitivity and specificity at sites absent from the array; errors at these sites can be reduced only through improved imputation. The Metabochip is absent from this plot, as it is not a genome-wide array. Plotted are 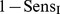 and 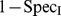, the sum of which equals the number of sites where (1) the gold-standard or called genotype is non-reference and (2) the gold-standard and called genotypes disagree. Normalized values (defined in Materials and Methods) are plotted to show visual trends; actual values are given in Figure S16(**b**) To assess the reduction in erroneous genotype cluster locations afforded by joint genotype calls with sequence data (relative to calls based on array data alone), we measured sensitivity and specificity at sites on the array. Red bars correspond to 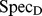 and 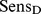, measured from calls without haplotype phasing; blue bars correspond to 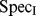 and 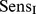, measured from joint calls. As described in Materials and Methods, these experiments used 82 additional unrelated samples, absent from our other experiments, to inform cluster locations.

We first asked if two specific error modes were resolved by joint calls. First, we examined sites where low coverage sequencing provided insufficient data to confidently call genotypes post imputation. We hypothesized that joint calls, made with addition of a high density SNP array, might significantly improve sensitivity at these sites by providing high quality genotypes to better identify haplotypes and inform imputation. We observe that joint calls do have higher 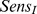 than calls from low coverage sequencing alone (Figure 2a), particularly for the lowest sequence coverages considered (.5× and 1×). However, the improvement in imputation quality is minimal when sequence coverage is 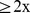 — evinced by almost undetectably larger joint call 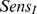 values at sites absent from the array, where any increases are due solely to improved imputation (Figure 4a, Figure S16).

We also examined sites where SNP arrays produce incorrect genotype calls due to erroneous or poorly resolved genotype cluster locations. We hypothesized that joint calls, informed within our framework by low coverage sequencing data in the same samples, might increase sensitivity through improved cluster locations. Our results show that joint calls do reduce incorrect genotype calls, with up to 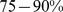 reductions in false negatives (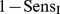, Figure 4b, Figure S17) across all minor allele frequencies (Figures S18). As previously reported, haplotype phasing [25] further increases sensitivity (Figure 4b, Figure S19). Thus, joint calls do reduce genotype errors due to improved cluster locations, although we also find that additional sequence data on the test sample has a significant impact (Figure S20).

To identify additional error modes, we searched for SNPs at which array data frequently disagreed with 4× sequencing data (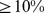 of samples with different genotypes). On the Metabochip — a custom array with many low frequency or unvalidated SNPs — we identified 3,262 such disputes. Analysis of these SNPs revealed two error modes that suggested altered analytical procedures for array or sequence genotyping.

First, 639 SNPs were called polymorphic in analysis of sequence data but monomorphic in analysis of the same samples on the Metabochip. In sequencing studies that genotype SNPs called from sequence data on a custom array and require polymorphic array genotypes to “validate” SNPs, these 639 SNPs would be flagged as false-positives in the original sequence-based discovery. However, joint calls of these 639 SNPs predict that 331 are actually polymorphic, while only 181 are monomorphic (127 are no-calls; Figure S21). Joint calls thus partially address false-negative genotype calls, a known SNP array error mode [21].

**Figure 5:**
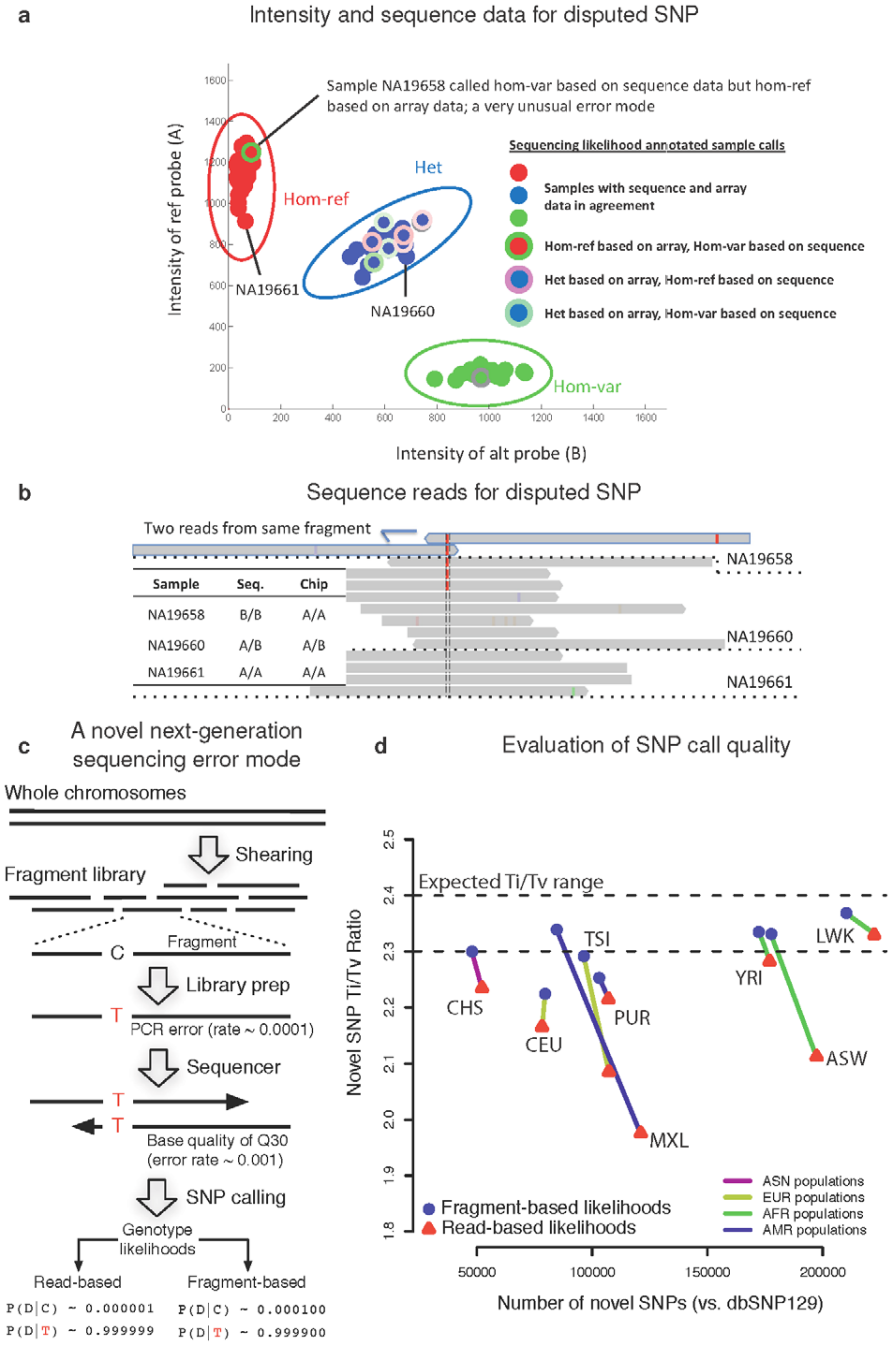
A novel next-generation sequencing error mode. (**a**) We identified a novel error mode based on visual examination of disputed SNPs. As shown in the cluster plot, one of the samples is called homozygous reference (Hom-ref) based on analysis of array data but homozygote non-reference (Hom-var) based on analysis of sequence data (shown by the sample outlined in green within the red cluster). This unusual error mode contrasts with the more common error mode, due to low sequence coverage, of samples called heterozygous (Het) based on array data but homozygous reference or non-reference based on sequence data (shown by samples outlined in pink or green within the blue cluster). (**b**) Inspection of the sequence reads in the Integrated Genomics Viewer (IGV) [54] shows that the sample in question has only two reads that cover this SNP, and these reads are pairs sequenced from the same underlying DNA fragment. (**c**) This error mode is introduced in the shearing and library preparation stage of next-generation sequencing and as a result is reflected in both reads from the same DNA fragment. Depending on protocol details, the error rate is around 1/10,000. During genotype calling, independent treatment of reads (read-based) results in much more confident (here 100×) non-reference genotype calls than analysis at the fragment level (fragment-based). (**d**) To account for these effects, which can be large for low coverage sequencing projects like the 1000G Project, we implemented a fragment based genotyping algorithm in the Unified Genotyper of the Genome Analysis Toolkit (GATK). Use of this new caller has a significant impact on SNP call quality, shown by a smaller number of novel SNP calls and a higher Transition∶Transversion ratio (proxies for accuracy [27]). The effect is pronounced for populations such as MXL and ASW, which have a higher fraction of newer Illumina sequencing data with longer reads (e.g., AWS data is 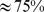 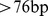 reads, while YRI has less than 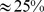), which results in greatly increased rate of overlapping reads and associated errors. Abbreviations are as defined in the 1000G Project.

Second, through visual examination of a number of disputed SNPs, we identified a recurrent sequencing error mode caused by PCR errors in the library preparation stage of next-generation sequencing [48] (Figure 5ab). Specifically, overlapping paired-end reads both reflect the same PCR error, and assumed independence of the read error rates leads to over-confident non-reference genotype calls (Figure 5c). This error mode is increasingly common given the growing length of sequencing reads (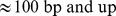) relative to standard library sizes (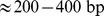). Based on this observation, we developed a new sequence genotype likelihood calculation that properly accounts the dependency of overlapping read pairs from the same DNA fragment. Briefly, the new method models errors at the fragment construction stage separately from errors during the sequencing stage, and it thus counts fragment errors only once for each read pair. (Figure S22a). Experiments showed that this new method improves both sequence genotyping accuracy and SNP site discovery (Figure 5d, Figure S22b); as a result, it has been incorporated into the Unified Genotyper of the Genome Analysis Toolkit (GATK) [27] and applied to many sequencing experiments including the production phase of the 1000 G Project.

Given the improvements that followed discovery of this novel sequencing error mode, further analysis of disputed SNPs might suggest additional unrecognized error modes and additional improvements to genotyping algorithms. When gold-standard genotypes are unavailable to serve as a filter between array and sequence errors, joint calls can serve as a proxy — of the disputed SNPs on Metabochip for which our test sample genotype is in disagreement, 85.7% of joint calls are consistent with gold-standard genotypes. Filtering SNPs with joint calls that closely match calls from sequence data as apparent array errors, and the remainder as apparent sequence errors (Figure S23a), classifies 70.5% of the disputed SNPs on the Metabochip as apparent sequence errors. Based on tests of enrichment for 18 potential error modes, this filtration strategy does in fact produce two distinct classes of SNPs: apparent array errors are enriched for DNA flanking regions of low complexity and flanking regions that occur multiple times in the genome (Figure S23b; Figures S24, S25), while apparent sequence errors are enriched for properties such as DNA strand bias or flanking homopolymer runs (Figure S23c). However, given that only 61% of the apparent array errors and 49% of the apparent sequence errors are characterized by one of the potential error modes we considered (Figure S26), analysis of the remaining SNPs may provide a rich source of novel error modes.

## Discussion

While high coverage sequencing remains the most accurate genotyping technology available, combinations of less comprehensive technologies can increase study efficiency and enable more samples to be genotyped. To this end, we developed a statistical framework that combines data from sequencing and array-based genotyping platforms, as well as imputation, to produce an integrated estimate of genetic variation within each individual. In this study we applied the framework to quantify the efficiency and power of strategies for data collection that include one or both of these technologies. As the pace of sequencing or other genotyping technology development quickens [48], [49], this work provides a foundation to call genotypes for samples analyzed on one or more new technologies — particularly those samples with prior data already available.

Our experiments with this framework measure the number of variant sites assayed in a single European individual and closely model the scenario where 1000 G Project data is used as a reference panel for imputation. The strategies we considered will likely assay fewer variant sites in non-European ethnicities, particularly those with less complete reference panels available (Text S1). The 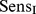 and 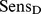 metrics we used in our experiments also have limitations. In some cases the total number of variant sites assayed across all individuals, a metric influenced far more by rarer variants, may be more relevant; no strategy we considered is a good assay of very rare or private variants (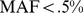), which are the focus of diagnostic studies [5]. Another relevant metric might be the power to associate disease alleles, which is influenced not only by sensitivity and specificity but by the total study size, the number of samples genotyped at each disease allele, and the disease architecture [35]. Further work is needed to fully understand the performance of each data collection strategy under these alternate metrics. Further work is also needed to quantify the ability of each strategy to assay small insertions or deletions [50] as well as larger structural variants [51], [52], which our experiments do not directly measure.

In addition, our experiments provide a snapshot in time of the quantitative performance of each strategy. As technologies improve and reference panel sizes increase, the absolute and relative values of our metrics will change. Higher sequence coverage or improved algorithms for read mapping and variant calling, which occur continuously, would increase sequencing performance relative to SNP arrays — not only through increased coverage but also through increased imputation performance due to fewer genotyping errors. Reduction in the already small error rates for SNP arrays will improve sensitivity and specificity only slightly, implying that the relatively slower increase in array density will offer the most significant improvements in array performance. Larger reference panels, which will soon have more than ten times as many samples as the reference panel used in our experiments, will improve the performance of all strategies — for lower frequency variants in particular.

Nonetheless, our results suggest that, for studies that characterize European samples and use the 1000 G reference panel for imputation, low coverage 1× sequencing assays a similar number of sites in each individual as the 1 M SNP arrays used for GWAS, while 2× sequencing out-performs even the highest density arrays currently available. This confirms low coverage sequencing, although of depth even lower than previously suggested or used [10], [37], as an attractive genotyping strategy relative to SNP arrays for samples characterized *de novo*. Based on the approximately 2.75 M non-reference genotypes in our European test sample, our metrics translate to about 300 k false negative and 15 k false positive genotypes per genome for 1× sequencing or 1 M SNP arrays, 200 k false negative and 8 k false positive genotypes for 2× sequencing, and 100 k false negative and 4.5 k false positive genotypes for 4× sequencing with a 2.5 M SNP array.

For studies with samples previously genotyped on genome-wide SNP arrays, addition of low coverage sequencing substantially improves performance — for lower frequency sites in particular. As a result, addition of 1× or even .5× sequence coverage provides more novel information than additional dense genome-wide array-based genotyping.

At the small number of sites where sequencing and array genotypes disagree, joint calls significantly reduce error rates. Array genotype errors due to incorrect or poorly resolved genotype cluster locations are reduced by as much as 75–90%. In contrast, sequence genotype errors due to poor imputation are also reduced but only by only a small amount.

Two error modes identified through analysis of disputed SNPs illustrate how our framework can suggest paths to improve technology-specific genotype calling algorithms. SNPs called monomorphic on a custom array may be false negatives of the array — sequencing false positive rates need thus be assessed through joint data analysis, rather than through use of array data as gold-standard. As another example, a novel class of sequencing errors inspired development of a sequence genotyping method that has improved SNP discovery and genotyping accuracy in the production phase of the 1000 G project.

At present, a framework for joint genotype estimation facilitates study design and reduces genotype errors. In the future, as hundreds of thousands of valuable samples already genotyped on genome-wide SNP arrays are targeted for further genotyping, we suggest that joint calls may be the most informative analytical strategy. At a minimum, such frameworks provide a principled approach to compare and combine the wider variety of data types that will inevitably be developed.

## Materials and Methods

### A statistical framework for joint genotype calls

We developed a statistical framework to compute genotypes for 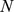 total samples at 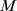 total biallelic SNPs, given intensity data 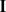 and sequence data 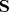. 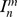 and 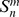 represent data for sample 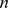 and SNP 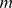, 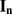 and 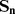 represent data for all SNPs for sample 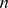, and 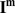 and 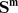 represent data for all samples for SNP 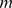; missing values are allowed for any 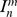 or 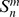. For each sample 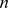 and SNP 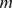, we estimate 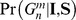, the posterior probability of the three genotypes, conditional upon intensity and sequence data for all samples and all SNPs.

The framework uses a naive Bayes model for sequence and intensity data — that is, 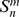 and 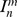 are conditionally independent given 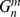. Thus, dependencies — between sequence and intensity data, across samples, or across SNPs — occur only because true genotypes are unknown. Values for 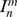 are 2-vectors that correspond to probe intensities for the two alleles of SNP 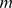 in sample 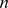; they are modeled under a mixture of 2-dimensional normal distributions, with a different mean (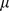), covariance (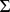), and prior probability (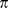) for each of the three genotype classes. The intensity model is thus
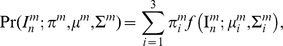
where 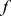 is the two-dimensional normal distribution. Sequence data is not modeled directly and is assumed to depend on parameters estimable without sample genotype knowledge. To allow any model for 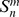 that satisfies this requirement, the framework accepts likelihood values 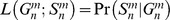 as input.

To call genotypes, the framework aims to maximize
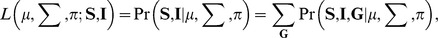
the likelihood of all parameters given observed sequence and intensity data for all samples and all SNPs. It employs an Expectation-Maximization (EM) algorithm which proceeds in iterations [53].

#### Initialization

Initial values for 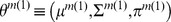 are estimated for each SNP. We implemented this step with the Birdseed algorithm [14], which estimates parameters separately for each SNP given intensity data.

#### E-step

Given current values of 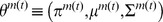, the E-step computes
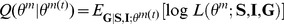
(1)

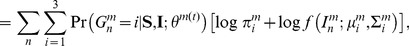
(2)which requires estimates of 
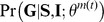
. To obtain these estimates for each SNP, we first compute
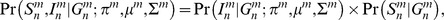
where values for 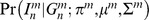 are given by the normal distribution density function, and values for 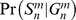 are given as input and remain constant throughout the algorithm by assumption. To estimate 
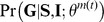
 then requires a haplotype phasing and imputation algorithm, because 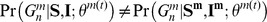 due to LD relationships between nearby SNPs. We implemented the phasing and imputation step with 20 iterations of the Beagle 3.3.0 algorithm [42], which accepts genotype likelihoods 
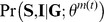
 as input and calculates values of 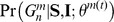 as output.

#### M-step

The parameters 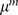, 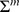, and 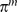 are updated through maximization of the equation that results from the E-step:
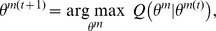
which yields
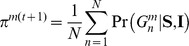


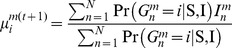


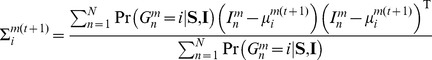



The E-step and M-step are iterated multiple times, and, upon termination, final values of 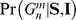 are returned as genotype calls. We used three iterations to obtain our experimental results. We did not explore the value of additional iterations, but we expect genotype accuracy to improve with additional iterations at the cost of added computational burden.

### Experimental data and procedure

For our experiments, we used our framework to call joint genotypes for combinations of intensity and read data from the Hapmap [9], [21] and 1000 G [10] projects (Figure S3). We tested five different density genome-wide SNP chips — the Affymetrix 100 K (Affy 100 k; 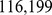 SNPs) and 500 K (Affy 500 k; 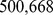 SNPs) GeneChip Mapping Sets, the Affymetrix Genome-Wide Human SNP Array 6.0 (Affy 6; 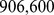 SNPs), the Illumina Human1M-Duo (Ilmn 1 M; 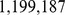 SNPs), and the Illumina HumanOmni2.5–8 (Omni 2.5, 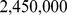 SNPs) — one custom SNP chip — the Illumina Cardio-Metabo Chip (Metabochip; 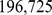 SNPs) — and four different levels of whole-genome sequence coverage — an average of one-half (.5×), one (1×), two (2×), and four (4×) reads aligned to each base in the genome.

Intensity data for the Affy 100 k and Affy 500 k arrays were downloaded from the International Hapmap Project website (http://hapmap.ncbi.nlm.nih.gov) and formatted for input with the Affymetrix Power Tools (APT) Software Package. To obtain intensity data for the Affy 6, Ilmn 1 M, Omni 2.5, and Metabochip arrays, we genotyped 180 Hapmap samples on each array using standard lab protocols; for the Omni 2.5 array, we also genotyped an additional 381 European samples from the 1000 G June 2011 Integrated Phase 1 Variant Release. For the Illumina arrays, raw data (in the form of IDAT files) was converted to normalized intensity data using a custom implementation IDATConverter of the Illumina normalization algorithm (algorithm details kindly provided by Illumina). Illumina normalized intensity data were then scaled by a factor of 2000 to be within the range expected for Birdseed allele intensity input files. Affymetrix raw intensity data were normalized with the quantile normalization method in the APT program apt-probeset-summarize.

Sequence data for the 180 Hapmap samples were downloaded from the 1000 genomes project Pilot 1 release, and data for the 381 European samples were downloaded from the Integrated Phase 1 Variant Release (http://www.1000genomes.org). Sequence data for each sample was roughly 4× coverage, and to approximate .5×, 1×, or 2× coverage, was down-sampled with the GATK [27] to randomly keep 12.5%, 25%, or 50% of the sequence reads.

We downloaded sequence and genotype data for our test sample (Hapmap sample NA12878) from the 1000 G Pilot 2 release. The genotype data was used as gold-standard data. We used the sequence data to approximate 4× coverage for our test sample: we kept reads from a sufficient number of read groups such that the average depth was as close as possible to 4× genome-wide, with read groups preferentially chosen to include those with the latest sequencing technology. We then further down-sampled this data with the GATK to approximate .5× (12.5%), 1× (25%), or 2× (50%) sequence coverage for our test sample.

Sequence likelihoods 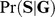 input to the joint calling framework were obtained from the downloaded or down-sampled sequence data with the Unified Genotyper (UG) module of the GATK. We ran the UG with its default arguments.

For each experiment, we jointly called genotypes for our test sample together with the 381 European samples. For all experiments we used 4× sequence coverage and Omni 2.5 intensity data for the 381 samples, while we varied the sequence and intensity data used for the test sample.

Because only our test sample had intensity data for the Affy 100 k, Affy 500 k, Affy 6, or Ilmn 1 M arrays, we needed to use additional samples to learn cluster locations for experiments with these arrays (a single sample cannot be clustered). These additional samples were 41 unrelated European and 41 unrelated African samples from among the 180 Hapmap samples genotyped on the arrays. For experiments that solely evaluated calls at sites on each array, we used joint calls from these 82 samples together with our test sample. For experiments that evaluated overall sensitivity and specificity, we used these 82 samples to obtain cluster locations that we then input to the joint calling framework.

Sensitivity and specificity were computed for each experiment as described in the main text. We report numbers computed only from SNPs on chromosome 20. We also omitted A/T or G/C SNPs when we computed our metrics to avoid possible DNA strand ambiguities.

### Normalized 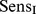 and 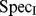


For our analysis of the improvement in imputation due to joint calls with array data, we analyzed 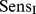 and 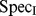 at sites absent from the array. Because each array contains a different set of sites, different SNPs were used to compute metrics for each array. Therefore, to show visual trends for this analysis, we plotted normalized rather than raw values of 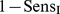 or 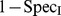. Normalized values are computed separately for each sequence coverage in three steps. First, raw joint call values (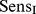 or 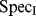) are computed for each array at sites absent from the array. These values are then divided by the corresponding value for calls from sequence data alone, computed at the same set of sites. Finally, these values are scaled by the corresponding value for calls from sequence data alone, computed over all sites. Actual values of 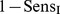 or 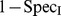 are given in Figure S16.

### Statistical analysis of discordant SNPs

We defined 18 potential error modes (5 binary, 13 continuous) and tested whether apparent sequence errors or apparent array errors were enriched for each. For the 5 binary error modes, we computed the fraction of apparent sequence errors and apparent array errors with the error mode and then assessed differences between the groups with Fisher's exact test; for the 13 continuous error modes, we used a Kruskal-Wallis one-way analysis of variance to test for differences between the apparent sequence error and apparent array error distributions. Nominally significant p-values (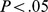) resulting from the test were taken as evidence that either apparent sequence errors or apparent array errors were enriched for the error mode.

To identify novel error modes, we filtered our apparent sequence errors and apparent array errors that were characterized by an error mode. We used one of two procedures for this; the goal was to flag disputes that could potentially (although not necessarily) be caused by an error mode. We considered a SNP as characterized by a binary error mode (value 0 or 1) if it had value 1 for the error mode. For continuous error modes, we used kernel density estimation as implemented in the R software package to fit separate probability density functions to SNPs not in dispute (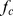), apparent sequence errors (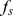), and apparent array errors (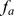). We then considered a SNP with value 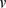 for the error mode as characterized if
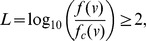
with 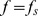 for apparent sequence errors and 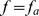 for apparent array errors.

## Supporting Information

Figure S1 **Sequence data and haplotype phasing inform SNP array cluster locations.** Our joint calling framework uses an iterative algorithm to estimate genotypes from sequence reads, SNP array intensities, and imputation. To call genotypes from intensity data requires estimation of cluster locations — the expected distribution of intensities given each genotype — which can be challenging for SNPs with low population frequencies or cluster locations that differ from prior expectations. Our framework estimates cluster locations conditional on not only SNP array intensity data, as do many array clustering algorithms, but also on sequence data and linkage disequilibrium relationships with nearby SNPs. As shown for this illustrative SNP, as more data informs the joint calls, the cluster locations typically improve. Each circle represents a sample, and the two axes represent probe intensities for each allele. Red, blue, and green colors correspond to the three genotypes (gray or black indicates no-calls): ovals represent cluster locations based on array calls, the outline of each circle represents the sequence calls, and the fill of each circle represents the joint call. (**a**) Cluster locations given only array data; no genotypes can be called. (**b**) Genotypes given only sequence data; most genotypes can be weakly called. (**c**) Genotypes obtained by multiplying SNP array and sequence genotype likelihoods; some genotypes can be called but the cluster locations do not change. (**d**) Genotypes given sequence and array data for this SNP only; most genotypes can be called and cluster locations begin to resolve. (**e**) Genotypes given sequence and array data for all SNPs; all genotypes can be called and cluster locations mostly resolve.
(PDF)

Figure S2 **Algorithm overview.** We implemented our framework in a Python program. The program accepts a set of partially overlapping intensity files, with SNP array data, and VCF files, with sequence genotype likelihoods. It initializes cluster locations and sample genotypes using the Birdseed algorithm, and then iteratively re-estimates sample genotypes and cluster locations. It uses the Beagle algorithm for phasing and imputation and a modified version of Birdseed to estimate cluster locations conditional on current genotype estimates, intensity data, and sequence data. After a number of iterations, the program produces a VCF file with posterior probabilities of all genotypes for all input samples at all input sites.
(PDF)

Figure S3 **Experimental procedure.** Schematic of the procedure used for our experiments. Details are given in Materials and Methods.
(PDF)

Figure S4 **Sensitivity and specificity of data collection strategies: 41 sample European reference panel.** Shown is data analogous to Figure 2 but for a 42 European samples rather than 382 samples. As described in Text S1, this closely models the use of a 41 European sample reference panel for imputation (just as our main experiments closely model the use of a 381 European sample reference panel). While the test sample remains the same as in Figure 2, we used different sequence data for this experiment — therefore, the SensD values differ. (**a**) Sensitivity of calls. (**b**) Specificity of calls. (**c**) SensI by variant frequency. (**d**) SensI for four sequence coverages. (**e**) SensI for four array densities.
(PDF)

Figure S5 **Sensitivity and specificity of data collection strategies: 41 sample African reference panel.** Shown is data analogous to Figure S4 but for an African reference panel rather than a European reference panel. (**a**) Sensitivity of calls. (**b**) Specificity of calls. (**c**) SensI by variant frequency. (**d**) SensI for four sequence coverages. (e) SensI for four array densities.
(PDF)

Figure S6 **Sensitivity and specificity of data collection strategies: no reference panel.** Shown is data analogous to Figures S4, S5 but absent a reference panel — all samples were sequenced to the depth and genotyped on the array referenced in the table. (**a**) 42 European samples sequenced. (**b**) 42 African samples sequenced.
(PDF)

Figure S7 **Impact of reference panel on sensitivity and specificity.** We constructed a reference panel in three different ways: from 4× sequence data (Seq panel), from 4× sequence data and the array data used to genotype the test sample (Seq and array panel), and from 4× sequence data and Omni 2.5 data (Seq and Omni panel). We then assessed sensitivity and specificity when the test sample was called with all combinations of sequence and array data. (**a**) a 381 European sample reference panel; (**b**) a 41 European sample reference panel; and (**c**) a 41 African sample reference panel. We find that the use of array data on top of 4× sequence data to build the reference panel has a small but significant effect on sensitivity for low coverage sequencing or small reference panels.
(PDF)

Figure S8 **Sensitivity gains from investment.** Shown are SensI values for all combinations of array and sequence data. Points are colored according to the array data collected and labeled with the sequence data collected. The x-axis plots SensD, a measure of genotyping investment intrinsic to a technology. SensD correlates, though not strictly, with cost. (**a**) 381 European sample reference panel. (**b**) 41 European sample reference panel. (**c**) 41 African sample reference panel.
(PDF)

Figure S9 **Specificity with and without imputation.** Shown is data analogous to Figure 2b but with SpecD in addition to SpecI. (**a**) 381 European sample reference panel. (**b**) 41 European sample reference panel. (**c**) 41 African sample reference panel.
(PDF)

Figure S10 **Impact of no-call threshold on sensitivity and specificity without imputation.** Shown are SensD and SpecD values, as computed in Figure 2a and Figure S9a, for different Phred-scaled genotype quality thresholds; if the genotype quality is X, the posterior probability of the most likely genotype is 1–10^−X^. For our main analysis, we called genotypes only at sites where the genotype quality exceeded 10. (**a**) No genotype quality thresholds; calls at all sites where most likely genotype probability exceeds 33.3%. (**b**) Genotype quality threshold of 10: calls at sites where the most likely genotype probability exceeds 90%. (**c**) Genotype quality threshold of 20: calls at sites where the most likely genotype probability exceeds 99%.
(PDF)

Figure S11 **Impact of no-call threshold on sensitivity and specificity with imputation.** Shown are numbers analogous to Figure S10 but for SensI and SpecI rather than SensD and SpecD. (**a**) No genotype quality thresholds. (**b**) Genotype quality threshold of 10. (**c**) Genotype quality threshold of 20.
(PDF)

Figure S12 **Sensitivity and specificity at heterozygous and homozygous variants.** Shown are data analogous to Figure 2ab but with sensitivity and specificity computed separately for variants at which the test sample has a heterozygous and homozygous genotype. (**a**) Heterozygous genotypes. (**b**) Homozygous non-reference genotypes.
(PDF)

Figure S13 **Sensitivity and specificity in coding regions.** Shown are data analogous to Figure 2ab but broken into metrics for coding and noncoding variants. (**a**) Coding variants. (**b**) Noncoding variants.
(PDF)

Figure S14 **Sensitivity and specificity by minor allele frequency: 381 European sample reference panel.** Shown are data analogous to Figure 2c but with SpecI in addition to SensI.
(PDF)

Figure S15 **Impact of prior array data on sensitivity: 381 sample European reference panel.** Shown are data analogous to Figure 3 but with an additional array (Affy 500 k). (**a**) All variants. (**b**) Variants with minor allele frequency (MAF) between .5 and 5%. (**c**) Variants with MAF>5%.
(PDF)

Figure S16 **Sensitivity and specificity at sites not on the array: actual values.** Shown is data analogous to Figure 4a but with actual values rather than normalized values. Calls based on sequence data (Seq) calls are plotted in red, joint (Joint) calls are plotted in blue. The red bars differ in size because the sites analyzed depend on the array. (**a**) 381 European sample reference panel. (**b**) 41 European sample reference panel. (**c**) 41 African sample reference panel.
(PDF)

Figure S17 **Impact of additional sequence data on sensitivity and specificity at sites on the array.** We evaluated joint calls from sequence and array data when different fractions of samples had sequence data available; as in Figure 4b, we called genotypes for a batch of 83 samples. We computed calls with (blue) and without (red) haplotype phasing. For each SNP array, we tested scenarios where no samples had sequence data (0%), one sample had high coverage sequence data (1%), two samples had high coverage sequence data (2%), 10%–50% of samples had low coverage sequence data, all but the test sample had low coverage sequence data (99%), and all samples had low coverage sequence data (100%). The test sample had sequence data only in the final case.
(PDF)

Figure S18 **Sensitivity and specificity at sites on the array by allele frequency.** Sensitivity and specificity at sites on each SNP array as a function of minor allele frequency. Joint calls were made in the same manner as described in Figure 4b. Results are stratified by SNP array and different colored lines represent different data combinations: joint call SensI (blue), joint calls SensD (cyan), array call SensI (green), and array call SensD (red). (**a**) Sensitivity. (**b**) Specificity.
(PDF)

Figure S19 **Impact of sequence data and phasing on sensitivity and specificity for sites on the array.** We computed joint calls for 83 samples (as in Figure 4b) but in four different ways: based on array data (Array only), based on array data with haplotype phasing (Array+Phasing), based on array data and sequence data without haplotype phasing (Array+Seq), and based on array data and sequence data with haplotype phasing (Joint).
(PDF)

Figure S20 **Impact of new clusters and joint likelihoods on sensitivity and specificity at sites on the chip.** At sites on the SNP array, joint calls have higher sensitivity and specificity than array calls for two reasons: genotype likelihoods for the test-sample are computed from both sequence and array data, and genotype cluster locations are computed based on sequence data and haplotype phasing (Figure 1, Figure S1). To quantify these two contributions, we compared sensitivity and specificity for array calls (Array only), for calls made from the joint likelihoods without cluster re-estimation (Likelihoods only), for calls made from array likelihoods but with cluster locations re-estimated from all data (Cluster only), and for joint calls (Joint). We did not use haplotype phasing to compute any genotype calls for this experiment. Joint calls were made in the same manner as described in Figure 4b.
(PDF)

Figure S21 **Analysis of SNPs initially classified as “false positive”.** We identified 639 SNPs on the Metabochip polymorphic based on sequence data but monomorphic based on array data. Based on the joint calls, 127 SNPs are no-called and therefore unresolved, 331 SNPs are polymorphic, and 181 SNPs are monomorphic. The left plots show calls based on array data (ovals represent genotype classes) and sequence data (outlines of circles represent genotype classes); the right plots show joint calls with colors and symbols as defined in Figure S1.
(PDF)

Figure S22 **Genotype likelihoods of fragment-based calling.** (a) Mathematical formalism for fragment-based (rather than read-based) SNP calling. The likelihood of a read pair given a hypothesized genotype GTAB with alleles A and B is calculated via a two-stage inference that weights the probability of each read independently by the probability of a PCR (or other) error occurring in the sequenced DNA fragment. In the above equation, *p* refers to error rate of the fragment, *f* refers to the base in the fragment, *e* refers to the error rate in the read, and *b* refers to the base in the read. Thus, errors that occur during fragment construction are counted only once, while errors that occur during sequencing are counted independently. (b) Comparison of the SNP genotype likelihood quality for GATK [27] SNP calls at sites also on the Omni 2.5 array (chromosome 20 only). Ideally calibrated likelihoods would follow the diagonal line. Fragment-based likelihoods are more accurate at all confidence levels, but the impact is most important for low confidence levels — which correspond to points with less certain likelihoods.
(PDF)

Figure S23 **Analysis of disputed SNPs.** As described in the text, we used our framework to resolve SNPs where array data widely disagreed with 4× sequencing data. We classify disputes as apparent sequence (or array) errors if the joint genotype calls disagree with calls based on sequence (or array) data. (**a**) Example disputed SNPs. The left plots show calls based on array data (ovals represent genotype classes) and sequence data (outlines of circles represent genotype classes); the right plots show joint calls with colors and symbols as defined in Figure S1. (**bc**) We asked which disputed SNPs were due to known or predicted error modes. Plotted are values for six potential error modes, each stratified across four classes of SNPs: agreed (sequence and array calls disagree for <10% of genotypes), disputed (sequence and array calls disagree for ≥10% of genotypes), apparent array errors, and apparent sequence errors. (**b**) Shown are the fraction of SNPs in each class (1) with a DNA flanking region that occurs at multiple genomic locations, (2) that lie within low complexity regions, and (3) that lie within 50 bp of another SNP. Apparent array errors have higher values for each of these metrics than apparent sequence errors. (**c**) Shown are distributions over SNPs for (1) the likelihood of forward/reverse DNA strand bias in sequence calls, (2) the length of a neighboring homopolymer run, and (3) the average (Phred-scaled) error rate of the reads supporting the sequence call (higher values signify more confident SNP calls). Apparent sequence errors have higher values for each of these metrics than apparent array errors.
(PDF)

Figure S24 **Properties of resolved SNPs with greater than 10% discordant genotypes.** Distributions for all potential error modes assessed on disputed SNPs, as in Figure 23bc.
(PDF)

Figure S25 **Properties of resolved SNPs with greater than 25% discordant genotypes.** The same data as in Figure S24 but for SNPs classified as disputed when array calls and sequence calls disagree for more than 25% of sample genotypes.
(PDF)

Figure S26 **Characterized disputed SNPs.** We fit separate distributions to each potential error mode for disputed and non-disputed SNPs, as well as for each class of apparent error mode. We considered a SNPs as characterized by an error mode according to one of two criteria: if the error mode was binary, it characterized a SNP if the SNP had value 1 for the error mode; if the error mode was continuous, it characterized a SNP if the log of the likelihood ratio (LOD) of the error mode distribution to the non-disputed distribution exceeded two. (**a**) SNPs with greater than 25% discordant genotypes. (**b**) SNPs with greater than 10% discordant genotypes.
(PDF)

Text S1 **Available as supporting supporting information are: (1) calculations that show how previously described genotype calling algorithms are similar in principle to the joint calling framework when one or more of sequence reads, SNP array intensities, or linkage disequilibrium is omitted; (2) sensitivity and specificity calculations with a 41 sample European reference panel and a 41 sample African reference panel; and (3) investigation of the increase of sensitivity with additional genotyping investment.**
(PDF)
