# Supplementary figures and images for: Efficiency and Power as a Function of Sequence Coverage, SNP Array Density, and Imputation
Source: PLoS Comput Biol. 2012 Jul 12;8(7):e1002604. doi: 10.1371/journal.pcbi.1002604 (PMC3395607; doi:10.1371/journal.pcbi.1002604)

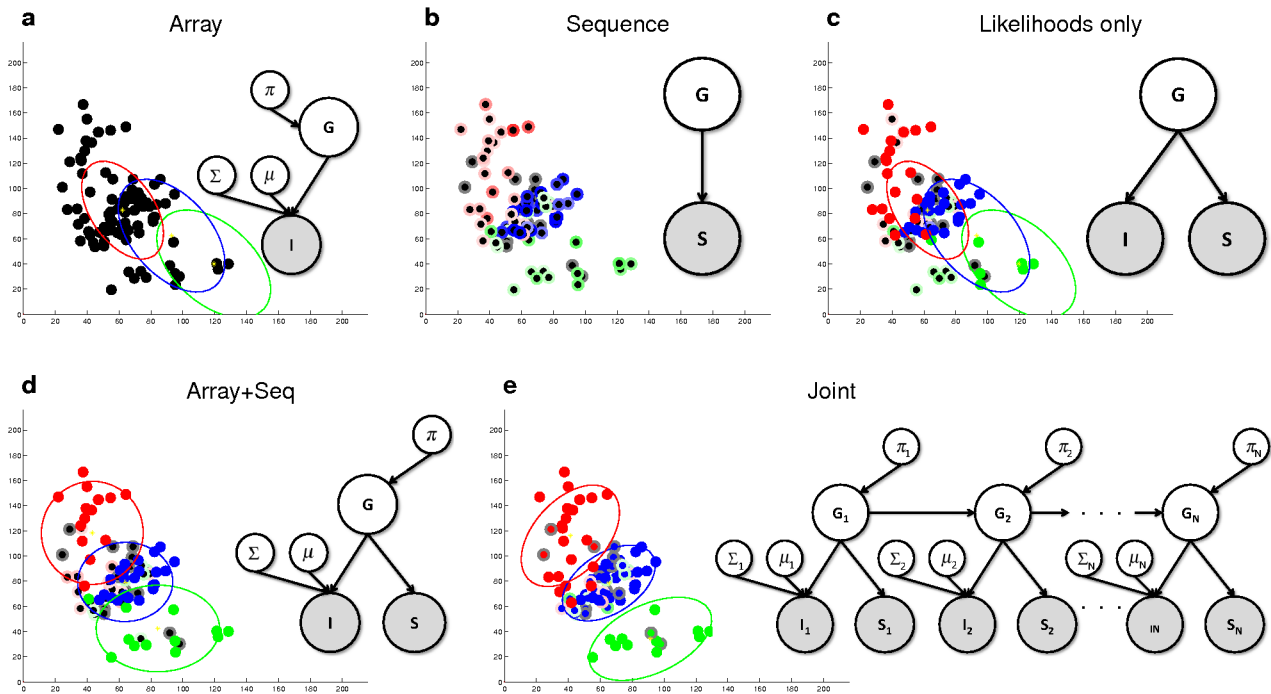

Supplement: Figure S1 — Sequence data and haplotype phasing inform SNP array cluster locations. Our joint calling framework uses an iterative algorithm to estimate genotypes from sequence reads, SNP array intensities, and imputation. To call genotypes from intensity data requires estimation of cluster locations — the expected distribution of intensities given each genotype — which can be challenging for SNPs with low population frequencies or cluster locations that differ from prior expectations. Our framework estimates cluster locations conditional on not only SNP array intensity data, as do many array clustering algorithms, but also on sequence data and linkage disequilibrium relationships with nearby SNPs. As shown for this illustrative SNP, as more data informs the joint calls, the cluster locations typically improve. Each circle represents a sample, and the two axes represent probe intensities for each allele. Red, blue, and green colors correspond to the three genotypes (gray or black indicates no-calls): ovals represent cluster locations based on array calls, the outline of each circle represents the sequence calls, and the fill of each circle represents the joint call. (a) Cluster locations given only array data; no genotypes can be called. (b) Genotypes given only sequence data; most genotypes can be weakly called. (c) Genotypes obtained by multiplying SNP array and sequence genotype likelihoods; some genotypes can be called but the cluster locations do not change. (d) Genotypes given sequence and array data for this SNP only; most genotypes can be called and cluster locations begin to resolve. (e) Genotypes given sequence and array data for all SNPs; all genotypes can be called and cluster locations mostly resolve. (PDF) [file pcbi.1002604.s001.pdf]

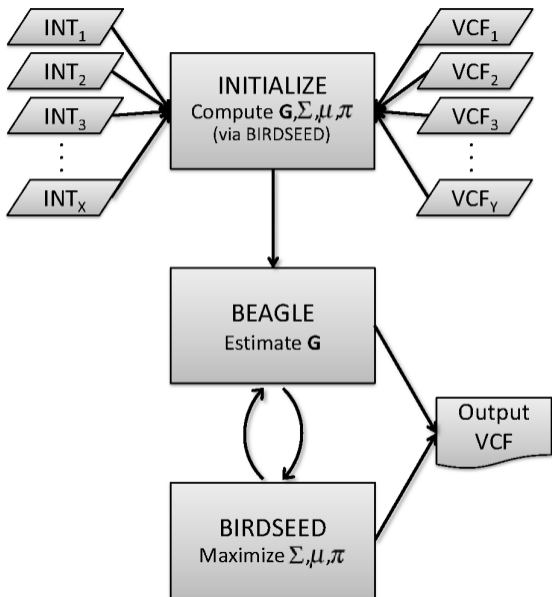

Supplement: Figure S2 — Algorithm overview. We implemented our framework in a Python program. The program accepts a set of partially overlapping intensity files, with SNP array data, and VCF files, with sequence genotype likelihoods. It initializes cluster locations and sample genotypes using the Birdseed algorithm, and then iteratively re-estimates sample genotypes and cluster locations. It uses the Beagle algorithm for phasing and imputation and a modified version of Birdseed to estimate cluster locations conditional on current genotype estimates, intensity data, and sequence data. After a number of iterations, the program produces a VCF file with posterior probabilities of all genotypes for all input samples at all input sites. (PDF) [file pcbi.1002604.s002.pdf]

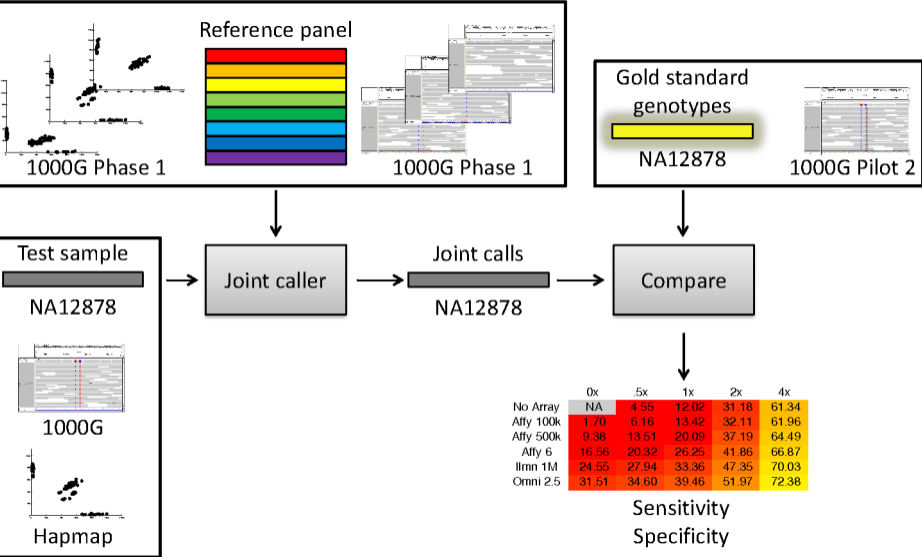

Supplement: Figure S3 — Experimental procedure. Schematic of the procedure used for our experiments. Details are given in Materials and Methods. (PDF) [file pcbi.1002604.s003.pdf]

# Sensitivity gains from investment

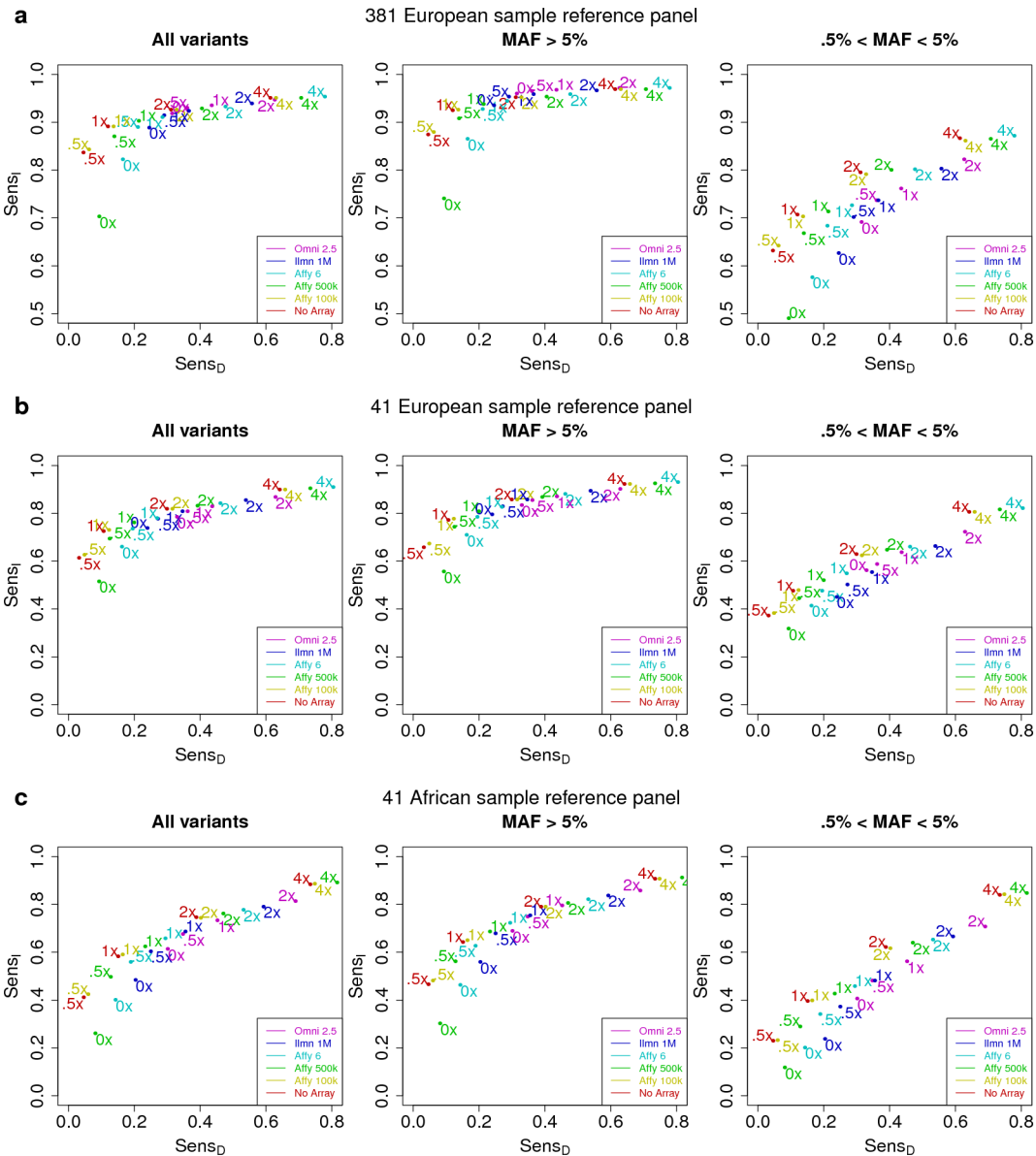

Supplement: Figure S8 — Sensitivity gains from investment. Shown are SensI values for all combinations of array and sequence data. Points are colored according to the array data collected and labeled with the sequence data collected. The x-axis plots SensD, a measure of genotyping investment intrinsic to a technology. SensD correlates, though not strictly, with cost. (a) 381 European sample reference panel. (b) 41 European sample reference panel. (c) 41 African sample reference panel. (PDF) [file pcbi.1002604.s008.pdf]

## Impact of prior array data on sensitivity

381 European sample reference panel

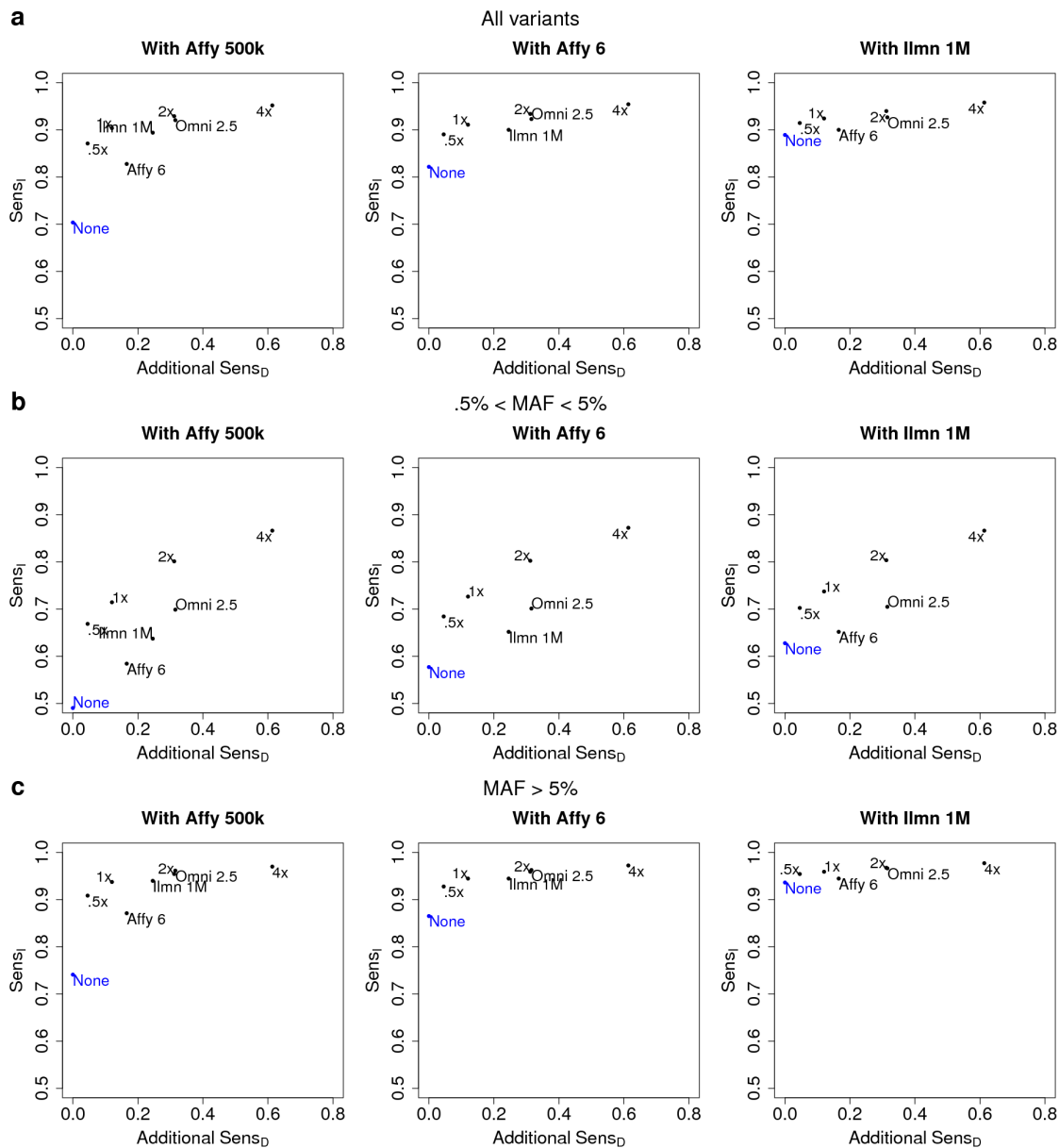

Supplement: Figure S15 — Impact of prior array data on sensitivity: 381 sample European reference panel. Shown are data analogous to Figure 3 but with an additional array (Affy 500 k). (a) All variants. (b) Variants with minor allele frequency (MAF) between .5 and 5%. (c) Variants with MAF>5%. (PDF) [file pcbi.1002604.s015.pdf]

Impact of additional sequence data on sensitivity and specificity at sites on array

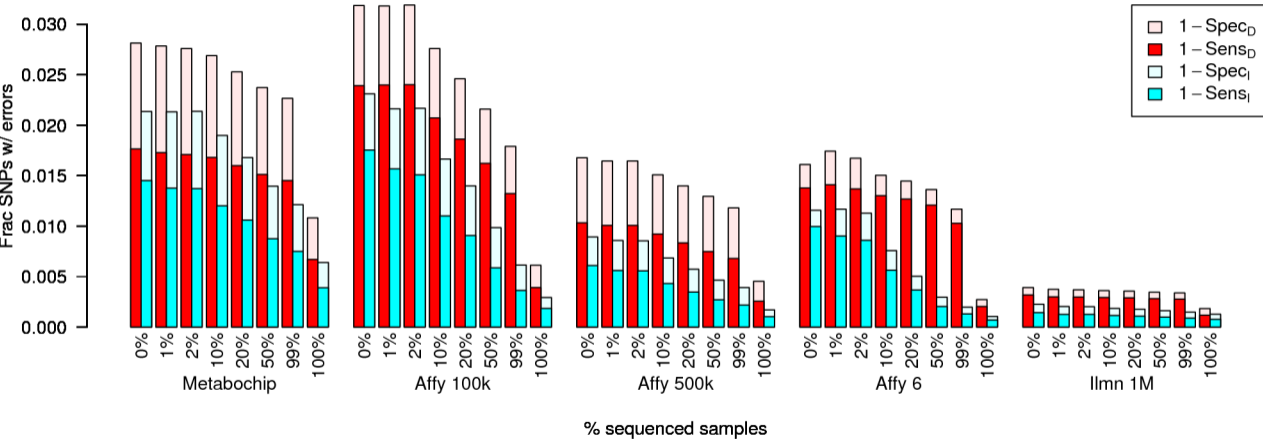

Supplement: Figure S17 — Impact of additional sequence data on sensitivity and specificity at sites on the array. We evaluated joint calls from sequence and array data when different fractions of samples had sequence data available; as in Figure 4b, we called genotypes for a batch of 83 samples. We computed calls with (blue) and without (red) haplotype phasing. For each SNP array, we tested scenarios where no samples had sequence data (0%), one sample had high coverage sequence data (1%), two samples had high coverage sequence data (2%), 10%–50% of samples had low coverage sequence data, all but the test sample had low coverage sequence data (99%), and all samples had low coverage sequence data (100%). The test sample had sequence data only in the final case. (PDF) [file pcbi.1002604.s017.pdf]

**a**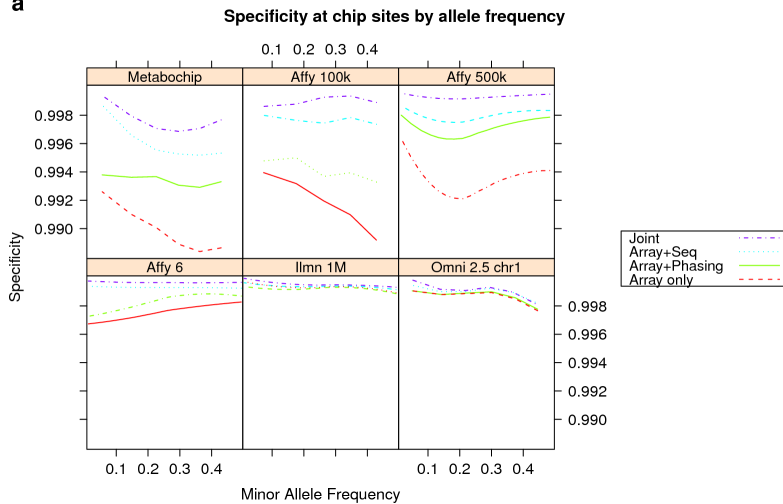**b**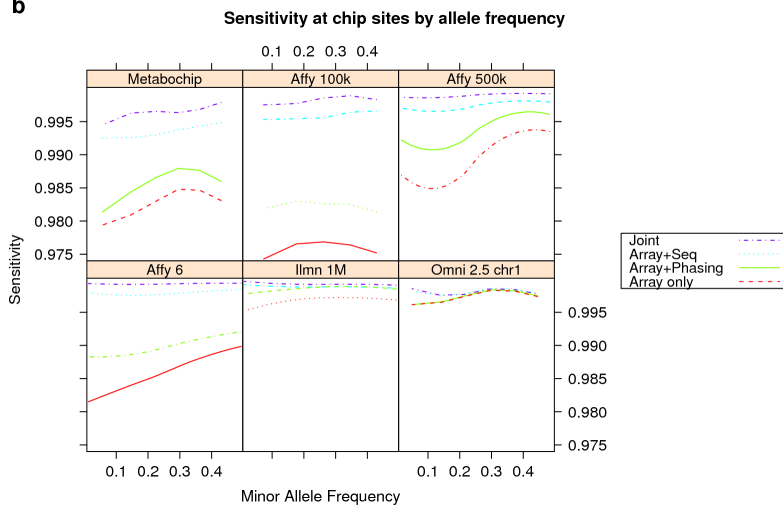

Supplement: Figure S18 — Sensitivity and specificity at sites on the array by allele frequency. Sensitivity and specificity at sites on each SNP array as a function of minor allele frequency. Joint calls were made in the same manner as described in Figure 4b. Results are stratified by SNP array and different colored lines represent different data combinations: joint call SensI (blue), joint calls SensD (cyan), array call SensI (green), and array call SensD (red). (a) Sensitivity. (b) Specificity. (PDF) [file pcbi.1002604.s018.pdf]

**Impact of sequence data and phasing on  
sensitivity and specificity for sites on the array**

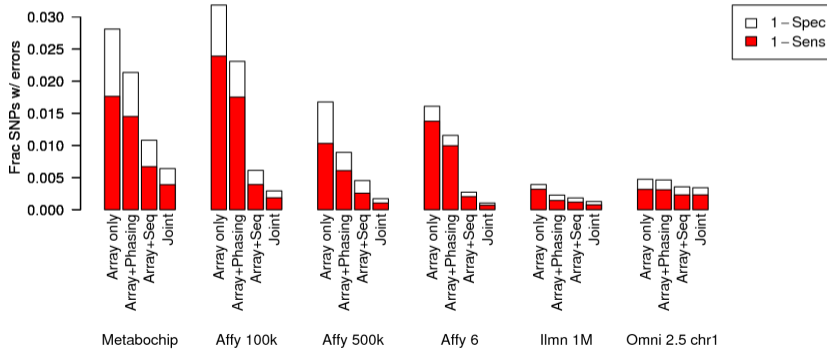

Supplement: Figure S19 — Impact of sequence data and phasing on sensitivity and specificity for sites on the array. We computed joint calls for 83 samples (as in Figure 4b) but in four different ways: based on array data (Array only), based on array data with haplotype phasing (Array+Phasing), based on array data and sequence data without haplotype phasing (Array+Seq), and based on array data and sequence data with haplotype phasing (Joint). (PDF) [file pcbi.1002604.s019.pdf]

# Analysis of SNPs initially classified as false positives

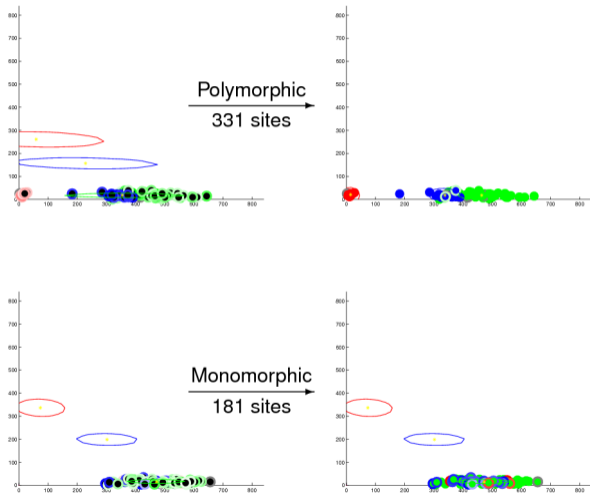

Supplement: Figure S21 — Analysis of SNPs initially classified as “false positive”. We identified 639 SNPs on the Metabochip polymorphic based on sequence data but monomorphic based on array data. Based on the joint calls, 127 SNPs are no-called and therefore unresolved, 331 SNPs are polymorphic, and 181 SNPs are monomorphic. The left plots show calls based on array data (ovals represent genotype classes) and sequence data (outlines of circles represent genotype classes); the right plots show joint calls with colors and symbols as defined in Figure S1. (PDF) [file pcbi.1002604.s021.pdf]

# Properties of resolved SNPs with $\geq 10\%$ discordant genotypes

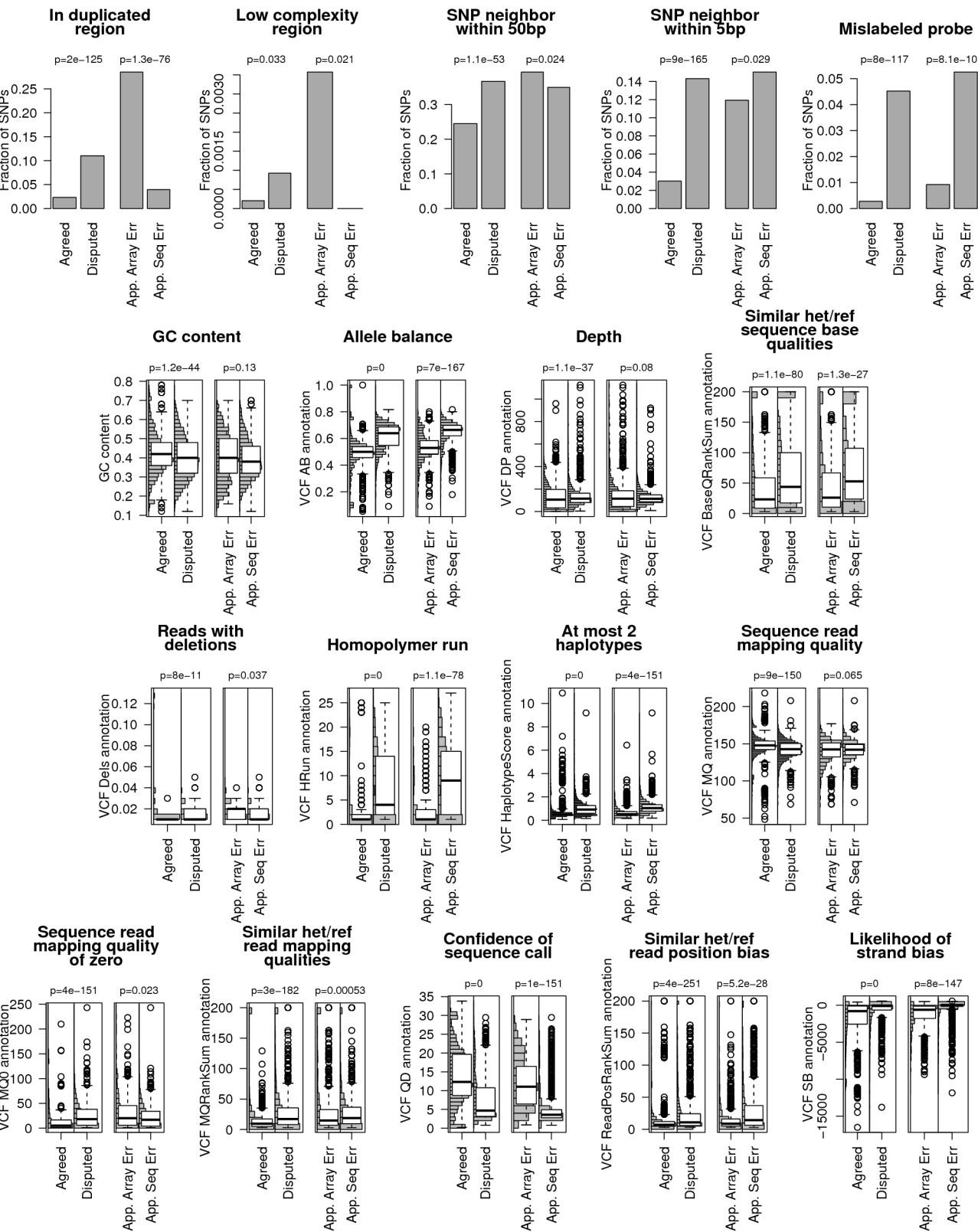

Supplement: Figure S24 — Properties of resolved SNPs with greater than 10% discordant genotypes. Distributions for all potential error modes assessed on disputed SNPs, as in Figure 23bc. (PDF) [file pcbi.1002604.s024.pdf]

# Properties of resolved SNPs with $\geq 25\%$ discordant genotypes

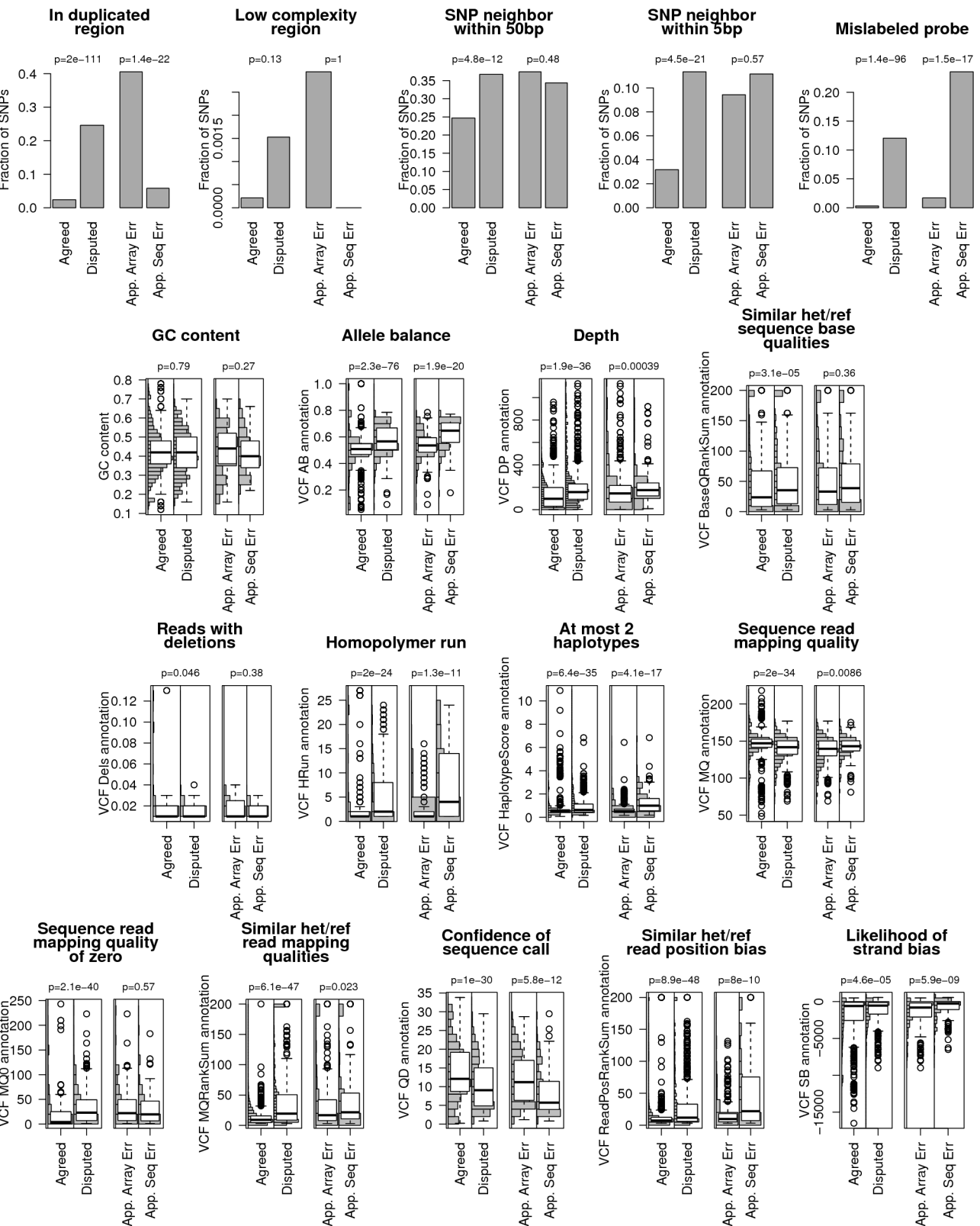

Supplement: Figure S25 — Properties of resolved SNPs with greater than 25% discordant genotypes. The same data as in Figure S24 but for SNPs classified as disputed when array calls and sequence calls disagree for more than 25% of sample genotypes. (PDF) [file pcbi.1002604.s025.pdf]
