# Supplementary material for: Efficiency and Power as a Function of Sequence Coverage, SNP Array Density, and Imputation
Source: PLoS Comput Biol. 2012 Jul 12;8(7):e1002604. doi: 10.1371/journal.pcbi.1002604 (PMC3395607; doi:10.1371/journal.pcbi.1002604)

Sensitivity and specificity of data collection strategies

41 European sample reference panel

a

Sens_D

	0x	.5x	1x	2x	4x
No Array	NA	3.22	10.68	29.92	64.18
Affy 100k	1.66	4.78	11.98	30.73	64.45
Affy 500k	9.31	12.19	18.82	36.09	66.86
Affy 6	16.27	18.82	24.86	40.74	69.18
Ilmn 1M	23.98	26.30	31.85	46.14	72.06
Omni 2.5	32.99	35.10	39.87	52.66	75.55

Sens_I

	0x	.5x	1x	2x	4x
No Array	NA	61.34	72.67	82.05	90.09
Affy 100k	15.30	62.74	73.12	82.08	90.06
Affy 500k	51.71	69.53	76.19	83.20	90.47
Affy 6	66.04	73.61	78.08	84.40	90.97
Ilmn 1M	73.86	77.75	80.91	85.60	91.66
Omni 2.5	78.55	80.86	82.97	86.94	92.18

b

Spec_I

	0x	.5x	1x	2x	4x
No Array	NA	98.48	98.84	99.15	99.46
Affy 100k	97.63	98.54	98.88	99.24	99.46
Affy 500k	98.27	98.82	98.98	99.18	99.41
Affy 6	98.86	98.95	99.19	99.27	99.46
Ilmn 1M	99.38	99.29	99.45	99.51	99.56
Omni 2.5	99.57	99.56	99.55	99.57	99.62

c

Sens_I for private variants

	0x	.5x	1x	2x	4x
No Array	NA	19.83	26.26	41.85	61.47
Affy 100k	3.36	20.02	26.50	42.92	60.81
Affy 500k	13.49	23.24	28.17	42.57	61.79
Affy 6	17.00	23.99	29.17	43.59	61.76
Ilmn 1M	18.50	24.04	29.89	44.22	60.97
Omni 2.5	20.41	26.96	32.56	44.83	63.03

Sens_I for $.5\% < \text{MAF} < 5\%$ variants

	0x	.5x	1x	2x	4x
No Array	NA	37.39	47.57	63.00	80.59
Affy 100k	11.34	38.33	48.04	62.52	80.58
Affy 500k	31.81	44.70	52.00	64.85	81.73
Affy 6	41.59	47.82	54.98	66.05	82.12
Ilmn 1M	45.20	50.37	55.38	66.48	82.31
Omni 2.5	56.24	58.89	63.71	72.27	84.58

Sens_I for $\text{MAF} > 5\%$ variants

	0x	.5x	1x	2x	4x
No Array	NA	65.84	77.39	85.78	92.27
Affy 100k	16.32	67.35	77.88	85.84	92.27
Affy 500k	55.69	74.33	80.90	86.89	92.59
Affy 6	70.97	78.61	82.71	88.09	93.12
Ilmn 1M	79.50	83.11	85.88	89.40	93.92
Omni 2.5	83.54	85.64	87.22	90.27	94.19

d

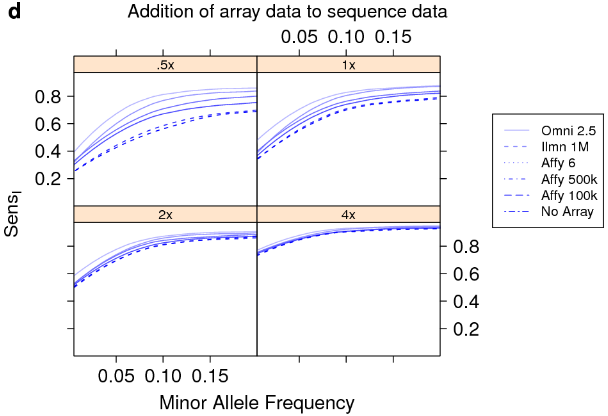

e

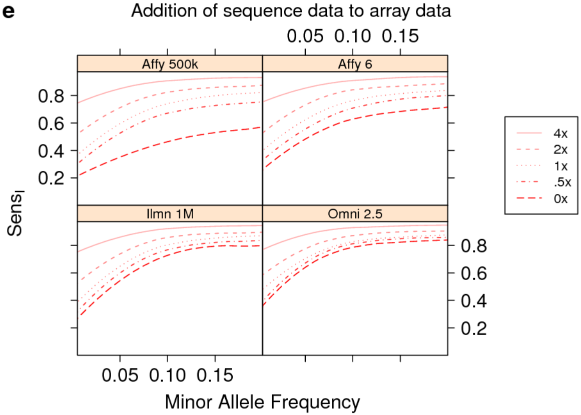

Supplement: Figure S4 — Sensitivity and specificity of data collection strategies: 41 sample European reference panel. Shown is data analogous to Figure 2 but for a 42 European samples rather than 382 samples. As described in Text S1, this closely models the use of a 41 European sample reference panel for imputation (just as our main experiments closely model the use of a 381 European sample reference panel). While the test sample remains the same as in Figure 2, we used different sequence data for this experiment — therefore, the SensD values differ. (a) Sensitivity of calls. (b) Specificity of calls. (c) SensI by variant frequency. (d) SensI for four sequence coverages. (e) SensI for four array densities. (PDF) [file pcbi.1002604.s004.pdf]
