# Supplementary material for: Efficiency and Power as a Function of Sequence Coverage, SNP Array Density, and Imputation
Source: PLoS Comput Biol. 2012 Jul 12;8(7):e1002604. doi: 10.1371/journal.pcbi.1002604 (PMC3395607; doi:10.1371/journal.pcbi.1002604)

Sensitivity and specificity of data collection strategies

41 African sample reference panel

a

$Sens_D$

	0x	.5x	1x	2x	4x
No Array	NA	4.64	15.14	38.87	73.47
Affy 100k	1.36	5.82	15.91	39.09	73.59
Affy 500k	8.20	12.38	21.78	43.16	75.15
Affy 6	14.31	18.20	26.96	46.90	76.70
Ilmn 1M	20.40	24.02	32.27	50.87	78.49
Omni 2.5	30.15	33.41	40.72	57.10	80.96

$Sens_I$

	0x	.5x	1x	2x	4x
No Array	NA	41.15	58.33	74.60	88.58
Affy 100k	5.29	42.56	59.03	74.52	88.68
Affy 500k	26.05	49.74	62.40	76.24	89.19
Affy 6	40.20	55.94	65.80	77.70	89.59
Ilmn 1M	48.52	60.55	68.66	79.22	90.25
Omni 2.5	61.43	67.72	73.29	81.34	90.99

b

$Spec_I$

	0x	.5x	1x	2x	4x
No Array	NA	97.46	98.14	98.73	99.24
Affy 100k	96.65	97.50	98.21	98.71	99.24
Affy 500k	97.99	97.95	98.16	98.81	99.21
Affy 6	98.07	98.27	98.54	98.86	99.26
Ilmn 1M	98.92	98.86	98.79	99.08	99.37
Omni 2.5	99.16	99.12	99.10	99.29	99.43

c

$Sens_I$ for private variants

	0x	.5x	1x	2x	4x
No Array	NA	18.66	27.84	46.15	69.15
Affy 100k	7.15	18.37	27.77	45.59	69.22
Affy 500k	13.53	19.91	29.20	46.72	69.45
Affy 6	15.57	21.13	28.65	47.36	69.64
Ilmn 1M	16.66	22.17	29.99	47.46	69.91
Omni 2.5	19.58	24.50	32.41	47.68	69.94

$Sens_I$ for .5% $<$ MAF $<$ 5% variants

	0x	.5x	1x	2x	4x
No Array	NA	23.00	39.62	62.20	84.06
Affy 100k	3.43	23.28	39.97	61.60	84.22
Affy 500k	11.81	29.12	42.90	63.95	84.73
Affy 6	20.29	34.29	45.90	65.36	85.00
Ilmn 1M	23.80	37.35	48.28	66.57	85.61
Omni 2.5	40.73	48.18	56.23	70.78	86.92

$Sens_I$ for MAF $>$ 5% variants

	0x	.5x	1x	2x	4x
No Array	NA	46.61	64.21	79.00	90.80
Affy 100k	5.82	48.32	65.02	79.04	90.90
Affy 500k	30.23	56.17	68.61	80.69	91.42
Affy 6	46.28	62.86	72.33	82.23	91.86
Ilmn 1M	56.01	68.00	75.40	83.89	92.55
Omni 2.5	68.89	74.86	79.60	85.77	93.25

d

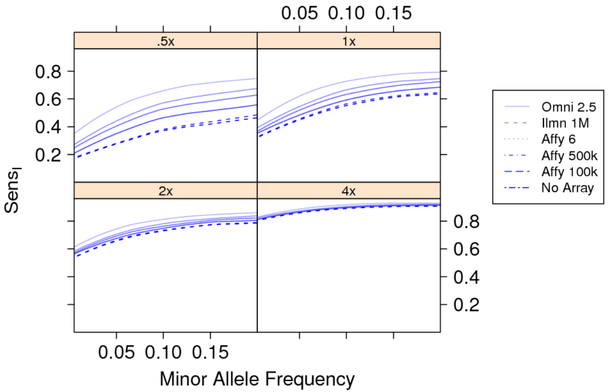

e

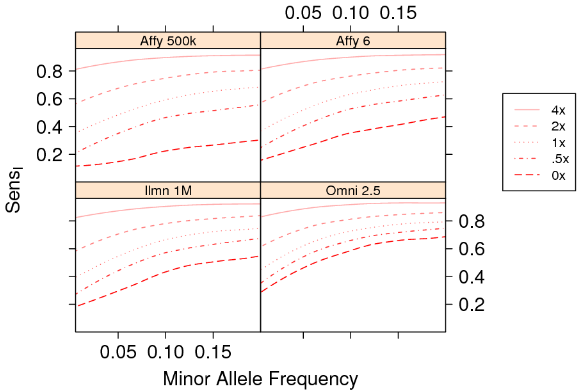

Supplement: Figure S5 — Sensitivity and specificity of data collection strategies: 41 sample African reference panel. Shown is data analogous to Figure S4 but for an African reference panel rather than a European reference panel. (a) Sensitivity of calls. (b) Specificity of calls. (c) SensI by variant frequency. (d) SensI for four sequence coverages. (e) SensI for four array densities. (PDF) [file pcbi.1002604.s005.pdf]
