# Supplementary material for: Efficiency and Power as a Function of Sequence Coverage, SNP Array Density, and Imputation
Source: PLoS Comput Biol. 2012 Jul 12;8(7):e1002604. doi: 10.1371/journal.pcbi.1002604 (PMC3395607; doi:10.1371/journal.pcbi.1002604)
Supplement: Figure S6 — Sensitivity and specificity of data collection strategies: no reference panel. Shown is data analogous to Figures S4, S5 but absent a reference panel — all samples were sequenced to the depth and genotyped on the array referenced in the table. (a) 42 European samples sequenced. (b) 42 African samples sequenced. (PDF) [file pcbi.1002604.s006.pdf]

Sensitivity and specificity of data collection strategies without a reference panel

a 42 European samples sequenced

$Sens_I$

	0x	.5x	1x	2x	4x
No Array	NA	3.25	14.84	62.09	88.41
Affy 100k	1.66	4.84	17.59	63.64	88.77
Affy 500k	9.37	12.49	28.12	68.17	89.41
Affy 6	16.39	19.34	36.57	71.93	89.94
Ilmn 1M	24.02	26.91	44.84	76.41	90.96
Omni 2.5	32.99	35.95	53.95	81.32	92.18

$Spec_I$

	0x	.5x	1x	2x	4x
No Array	NA	98.95	97.98	98.59	99.40
Affy 100k	99.88	99.18	98.37	98.69	99.50
Affy 500k	99.79	99.40	98.76	98.97	99.42
Affy 6	99.82	99.50	99.04	99.19	99.49
Ilmn 1M	99.88	99.75	99.22	99.30	99.56
Omni 2.5	99.87	99.73	99.32	99.47	99.62

b 42 African samples sequenced

$Sens_I$

	0x	.5x	1x	2x	4x
No Array	NA	4.66	15.68	48.49	86.36
Affy 100k	1.37	5.85	16.96	50.55	87.09
Affy 500k	8.22	12.53	23.86	55.59	88.06
Affy 6	14.44	18.54	29.89	59.87	88.82
Ilmn 1M	20.40	24.33	35.51	64.06	89.59
Omni 2.5	30.15	33.87	44.49	70.02	90.99

$Spec_I$

	0x	.5x	1x	2x	4x
No Array	NA	99.40	98.59	98.09	99.13
Affy 100k	99.65	99.35	98.76	98.29	99.16
Affy 500k	99.81	99.59	99.01	98.54	99.22
Affy 6	99.59	99.51	99.01	98.72	99.26
Ilmn 1M	99.95	99.78	99.30	98.89	99.37
Omni 2.5	99.86	99.76	99.29	99.08	99.43
