# Supplementary material for: Efficiency and Power as a Function of Sequence Coverage, SNP Array Density, and Imputation
Source: PLoS Comput Biol. 2012 Jul 12;8(7):e1002604. doi: 10.1371/journal.pcbi.1002604 (PMC3395607; doi:10.1371/journal.pcbi.1002604)

## Impact of reference panel on sensitivity and specificity

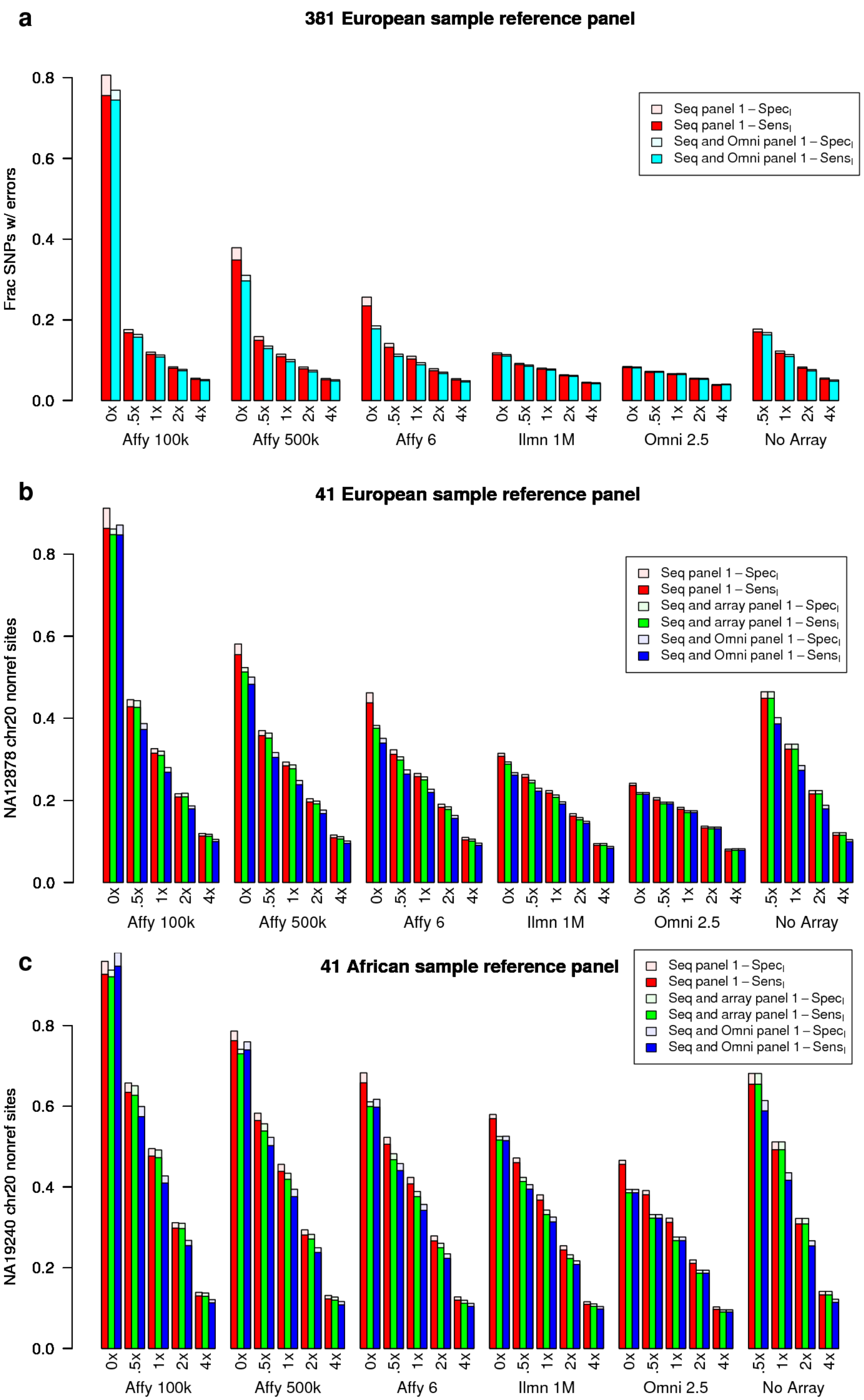

Supplement: Figure S7 — Impact of reference panel on sensitivity and specificity. We constructed a reference panel in three different ways: from 4× sequence data (Seq panel), from 4× sequence data and the array data used to genotype the test sample (Seq and array panel), and from 4× sequence data and Omni 2.5 data (Seq and Omni panel). We then assessed sensitivity and specificity when the test sample was called with all combinations of sequence and array data. (a) a 381 European sample reference panel; (b) a 41 European sample reference panel; and (c) a 41 African sample reference panel. We find that the use of array data on top of 4× sequence data to build the reference panel has a small but significant effect on sensitivity for low coverage sequencing or small reference panels. (PDF) [file pcbi.1002604.s007.pdf]
