# Supplementary material for: Efficiency and Power as a Function of Sequence Coverage, SNP Array Density, and Imputation
Source: PLoS Comput Biol. 2012 Jul 12;8(7):e1002604. doi: 10.1371/journal.pcbi.1002604 (PMC3395607; doi:10.1371/journal.pcbi.1002604)
Supplement: Figure S9 — Specificity with and without imputation. Shown is data analogous to Figure 2b but with SpecD in addition to SpecI. (a) 381 European sample reference panel. (b) 41 European sample reference panel. (c) 41 African sample reference panel. (PDF) [file pcbi.1002604.s009.pdf]

Specificity with and without imputation

a

381 European sample reference panel

	Spec$_D$					Spec$_I$				
	0x	.5x	1x	2x	4x	0x	.5x	1x	2x	4x
No Array	NA	98.97	99.10	98.32	98.44	NA	99.39	99.48	99.68	99.72
Affy 100k	99.88	99.24	99.19	98.42	98.49	97.53	99.33	99.48	99.66	99.70
Affy 500k	99.35	99.36	99.27	98.62	98.61	98.61	99.36	99.46	99.60	99.68
Affy 6	99.86	99.69	99.60	98.96	98.77	99.27	99.48	99.49	99.62	99.67
Ilmn 1M	99.94	99.82	99.69	99.17	98.91	99.63	99.70	99.70	99.74	99.78
Omni 2.5	99.80	99.77	99.73	99.24	99.04	99.75	99.78	99.75	99.80	99.83

b

41 European sample reference panel

	Spec$_D$					Spec$_I$				
	0x	.5x	1x	2x	4x	0x	.5x	1x	2x	4x
No Array	NA	98.94	98.74	98.16	98.11	NA	98.48	98.84	99.15	99.46
Affy 100k	99.88	99.26	98.88	98.24	98.17	97.63	98.54	98.88	99.24	99.46
Affy 500k	99.51	99.40	99.19	98.59	98.37	98.27	98.82	98.98	99.18	99.41
Affy 6	99.73	99.54	99.45	98.86	98.57	98.86	98.95	99.19	99.27	99.46
Ilmn 1M	99.93	99.83	99.61	99.09	98.75	99.38	99.29	99.45	99.51	99.56
Omni 2.5	99.87	99.79	99.62	99.28	98.91	99.57	99.56	99.55	99.57	99.62

c

41 African sample reference panel

	Spec$_D$					Spec$_I$				
	0x	.5x	1x	2x	4x	0x	.5x	1x	2x	4x
No Array	NA	99.33	98.89	98.09	98.00	NA	97.46	98.14	98.73	99.24
Affy 100k	99.53	99.40	99.00	98.17	98.08	96.65	97.50	98.21	98.71	99.24
Affy 500k	99.81	99.60	99.31	98.43	98.21	97.99	97.95	98.16	98.81	99.21
Affy 6	99.52	99.55	99.33	98.60	98.33	98.07	98.27	98.54	98.86	99.26
Ilmn 1M	99.95	99.83	99.53	98.83	98.49	98.92	98.86	98.79	99.08	99.37
Omni 2.5	99.86	99.84	99.59	99.04	98.66	99.16	99.12	99.10	99.29	99.43
