# Supplementary material for: Efficiency and Power as a Function of Sequence Coverage, SNP Array Density, and Imputation
Source: PLoS Comput Biol. 2012 Jul 12;8(7):e1002604. doi: 10.1371/journal.pcbi.1002604 (PMC3395607; doi:10.1371/journal.pcbi.1002604)
Supplement: Figure S10 — Impact of no-call threshold on sensitivity and specificity without imputation. Shown are SensD and SpecD values, as computed in Figure 2a and Figure S9a, for different Phred-scaled genotype quality thresholds; if the genotype quality is X, the posterior probability of the most likely genotype is 1–10−X. For our main analysis, we called genotypes only at sites where the genotype quality exceeded 10. (a) No genotype quality thresholds; calls at all sites where most likely genotype probability exceeds 33.3%. (b) Genotype quality threshold of 10: calls at sites where the most likely genotype probability exceeds 90%. (c) Genotype quality threshold of 20: calls at sites where the most likely genotype probability exceeds 99%. (PDF) [file pcbi.1002604.s010.pdf]

Impact of no-call threshold on sensitivity and specificity without imputation

381 European sample reference panel

a

Genotype quality threshold: None

$Sens_D$

	0x	.5x	1x	2x	4x
No Array	NA	15.08	27.85	50.08	74.98
Affy 100k	1.70	16.53	28.95	50.65	75.37
Affy 500k	9.40	22.77	34.07	53.92	76.78
Affy 6	16.60	28.70	39.03	57.42	78.55
Ilmn 1M	24.55	35.62	44.82	61.41	80.49
Omni 2.5	31.51	41.69	50.08	64.86	81.87

$Spec_D$

	0x	.5x	1x	2x	4x
No Array	NA	79.29	82.77	88.80	94.76
Affy 100k	99.76	81.09	83.80	89.19	94.89
Affy 500k	98.92	86.52	86.79	90.40	95.28
Affy 6	99.70	89.94	88.99	91.15	95.21
Ilmn 1M	99.94	92.59	91.32	92.63	95.84
Omni 2.5	99.80	94.25	92.87	93.86	96.61

b

Genotype quality threshold: 10

$Sens_D$

	0x	.5x	1x	2x	4x
No Array	NA	4.55	12.02	31.18	61.34
Affy 100k	1.70	6.16	13.42	32.11	61.96
Affy 500k	9.38	13.51	20.09	37.19	64.49
Affy 6	16.56	20.32	26.25	41.86	66.87
Ilmn 1M	24.55	27.94	33.36	47.35	70.03
Omni 2.5	31.51	34.60	39.46	51.97	72.38

$Spec_D$

	0x	.5x	1x	2x	4x
No Array	NA	98.97	99.10	98.32	98.44
Affy 100k	99.88	99.24	99.19	98.42	98.49
Affy 500k	99.35	99.36	99.27	98.62	98.61
Affy 6	99.86	99.69	99.60	98.96	98.77
Ilmn 1M	99.94	99.82	99.69	99.17	98.91
Omni 2.5	99.80	99.77	99.73	99.24	99.04

c

Genotype quality threshold: 20

$Sens_D$

	0x	.5x	1x	2x	4x
No Array	NA	3.44	8.66	20.92	42.10
Affy 100k	1.69	5.08	10.13	22.08	43.08
Affy 500k	9.29	12.46	17.03	28.08	47.32
Affy 6	16.45	19.37	23.50	33.65	51.16
Ilmn 1M	24.55	27.13	30.88	39.94	55.82
Omni 2.5	31.50	33.85	37.33	45.21	59.51

$Spec_D$

	0x	.5x	1x	2x	4x
No Array	NA	99.76	99.81	99.80	99.86
Affy 100k	99.88	99.80	99.82	99.83	99.86
Affy 500k	99.67	99.74	99.75	99.79	99.86
Affy 6	99.90	99.88	99.88	99.88	99.90
Ilmn 1M	99.94	99.93	99.91	99.88	99.90
Omni 2.5	99.83	99.87	99.87	99.85	99.89
