# Supplementary material for: Efficiency and Power as a Function of Sequence Coverage, SNP Array Density, and Imputation
Source: PLoS Comput Biol. 2012 Jul 12;8(7):e1002604. doi: 10.1371/journal.pcbi.1002604 (PMC3395607; doi:10.1371/journal.pcbi.1002604)
Supplement: Figure S11 — Impact of no-call threshold on sensitivity and specificity with imputation. Shown are numbers analogous to Figure S10 but for SensI and SpecI rather than SensD and SpecD. (a) No genotype quality thresholds. (b) Genotype quality threshold of 10. (c) Genotype quality threshold of 20. (PDF) [file pcbi.1002604.s011.pdf]

Impact of no-call threshold on sensitivity and specificity with imputation

381 European sample reference panel

a

Genotype quality threshold: None

$Sens_I$

	0x	.5x	1x	2x	4x
No Array	NA	92.42	94.41	96.11	97.26
Affy 100k	50.58	92.66	94.46	96.08	97.22
Affy 500k	85.98	93.62	94.81	96.11	97.16
Affy 6	91.14	94.25	95.08	96.31	97.32
Ilmn 1M	93.92	95.28	95.75	96.57	97.48
Omni 2.5	95.29	96.11	96.36	96.98	97.67

$Spec_I$

	0x	.5x	1x	2x	4x
No Array	NA	98.35	98.89	99.31	99.51
Affy 100k	88.86	98.38	98.96	99.31	99.51
Affy 500k	96.06	98.53	98.94	99.26	99.48
Affy 6	98.15	98.82	99.07	99.34	99.49
Ilmn 1M	99.18	99.36	99.47	99.50	99.63
Omni 2.5	99.53	99.57	99.60	99.62	99.70

b

Genotype quality threshold: 10

$Sens_I$

	0x	.5x	1x	2x	4x
No Array	NA	83.74	89.11	92.63	95.18
Affy 100k	25.56	84.30	89.22	92.61	95.07
Affy 500k	70.35	87.13	90.35	92.89	95.17
Affy 6	82.22	89.04	91.13	93.33	95.41
Ilmn 1M	88.96	91.50	92.46	94.02	95.81
Omni 2.5	91.93	92.99	93.57	94.72	96.10

$Spec_I$

	0x	.5x	1x	2x	4x
No Array	NA	99.39	99.48	99.68	99.72
Affy 100k	97.53	99.33	99.48	99.66	99.70
Affy 500k	98.61	99.36	99.46	99.60	99.68
Affy 6	99.27	99.48	99.49	99.62	99.67
Ilmn 1M	99.63	99.70	99.70	99.74	99.78
Omni 2.5	99.75	99.78	99.75	99.80	99.83

c

Genotype quality threshold: 20

$Sens_I$

	0x	.5x	1x	2x	4x
No Array	NA	59.23	71.15	80.39	88.73
Affy 100k	9.45	60.71	71.28	80.60	88.62
Affy 500k	43.68	66.28	73.95	81.74	89.17
Affy 6	58.68	70.64	76.33	82.82	89.68
Ilmn 1M	67.84	75.27	78.98	84.42	90.52
Omni 2.5	73.28	78.03	80.67	85.49	90.89

$Spec_I$

	0x	.5x	1x	2x	4x
No Array	NA	99.73	99.82	99.87	99.88
Affy 100k	99.09	99.70	99.77	99.85	99.85
Affy 500k	99.38	99.69	99.74	99.81	99.83
Affy 6	99.60	99.74	99.73	99.77	99.83
Ilmn 1M	99.84	99.86	99.85	99.88	99.89
Omni 2.5	99.90	99.90	99.88	99.91	99.90
