# Supplementary material for: Efficiency and Power as a Function of Sequence Coverage, SNP Array Density, and Imputation
Source: PLoS Comput Biol. 2012 Jul 12;8(7):e1002604. doi: 10.1371/journal.pcbi.1002604 (PMC3395607; doi:10.1371/journal.pcbi.1002604)
Supplement: Figure S12 — Sensitivity and specificity at heterozygous and homozygous variants. Shown are data analogous to Figure 2ab but with sensitivity and specificity computed separately for variants at which the test sample has a heterozygous and homozygous genotype. (a) Heterozygous genotypes. (b) Homozygous non-reference genotypes. (PDF) [file pcbi.1002604.s012.pdf]

Sensitivity and specificity at heterozygous and homozygous non-reference sites

381 European sample reference panel

a

Heterozygous variants

$Sens_I$

	0x	.5x	1x	2x	4x
No Array	NA	79.85	86.40	90.53	93.63
Affy 100k	21.50	80.44	86.62	90.46	93.53
Affy 500k	65.38	84.20	88.00	90.90	93.74
Affy 6	78.62	86.48	88.94	91.50	94.07
Ilmn 1M	86.29	89.17	90.34	92.21	94.43
Omni 2.5	89.89	90.98	91.64	92.98	94.78

$Spec_I$

	0x	.5x	1x	2x	4x
No Array	NA	99.55	99.62	99.79	99.84
Affy 100k	98.59	99.57	99.65	99.77	99.79
Affy 500k	98.60	99.39	99.52	99.65	99.75
Affy 6	99.34	99.54	99.57	99.69	99.71
Ilmn 1M	99.75	99.80	99.79	99.86	99.88
Omni 2.5	99.81	99.86	99.83	99.87	99.91

b

Homozygous variants

$Sens_I$

	0x	.5x	1x	2x	4x
No Array	NA	91.42	94.49	96.79	98.25
Affy 100k	33.61	91.95	94.39	96.86	98.12
Affy 500k	80.17	92.93	95.01	96.82	97.98
Affy 6	89.34	94.11	95.45	96.93	98.05
Ilmn 1M	94.25	96.12	96.68	97.60	98.53
Omni 2.5	95.98	96.97	97.37	98.18	98.72

$Spec_I$

	0x	.5x	1x	2x	4x
No Array	NA	99.12	99.21	99.47	99.48
Affy 100k	96.22	98.90	99.19	99.46	99.51
Affy 500k	98.62	99.31	99.37	99.50	99.55
Affy 6	99.14	99.39	99.34	99.49	99.59
Ilmn 1M	99.43	99.51	99.54	99.53	99.60
Omni 2.5	99.63	99.62	99.60	99.65	99.70
