# Supplementary material for: Efficiency and Power as a Function of Sequence Coverage, SNP Array Density, and Imputation
Source: PLoS Comput Biol. 2012 Jul 12;8(7):e1002604. doi: 10.1371/journal.pcbi.1002604 (PMC3395607; doi:10.1371/journal.pcbi.1002604)
Supplement: Figure S13 — Sensitivity and specificity in coding regions. Shown are data analogous to Figure 2ab but broken into metrics for coding and noncoding variants. (a) Coding variants. (b) Noncoding variants. (PDF) [file pcbi.1002604.s013.pdf]

Sensitivity and specificity of data collection strategies in coding regions

381 European sample reference panel

a

Coding variants

Sens_I

	0x	.5x	1x	2x	4x
No Array	NA	81.22	87.31	91.12	97.46
Affy 100k	15.23	81.98	86.29	92.39	97.21
Affy 500k	58.88	84.52	87.56	92.64	96.70
Affy 6	73.60	88.07	90.10	92.89	97.21
Ilmn 1M	91.88	91.88	93.65	94.67	96.95
Omni 2.5	93.65	94.92	94.67	95.43	97.97

Spec_I

	0x	.5x	1x	2x	4x
No Array	NA	99.69	99.42	100.00	100.[illegible]
Affy 100k	100.00	99.38	99.71	100.00	100.[illegible]
Affy 500k	99.57	99.70	99.71	99.73	99.[illegible]
Affy 6	99.66	99.71	99.72	99.73	99.[illegible]
Ilmn 1M	100.00	100.00	99.73	100.00	100.[illegible]
Omni 2.5	100.00	100.00	99.73	100.00	100.[illegible]

b

Noncoding variants

Sens_I

	0x	.5x	1x	2x	4x
No Array	NA	83.34	88.60	92.10	94.87
Affy 100k	26.74	83.86	88.70	92.09	94.76
Affy 500k	70.37	86.69	89.82	92.41	94.89
Affy 6	82.00	88.58	90.67	92.87	95.14
Ilmn 1M	88.42	90.93	91.87	93.52	95.55
Omni 2.5	91.38	92.50	93.13	94.38	95.93

Spec_I

	0x	.5x	1x	2x	4x
No Array	NA	99.41	99.49	99.68	99.73
Affy 100k	97.70	99.34	99.49	99.67	99.70
Affy 500k	98.64	99.38	99.48	99.61	99.69
Affy 6	99.29	99.50	99.50	99.63	99.68
Ilmn 1M	99.64	99.70	99.71	99.75	99.79
Omni 2.5	99.75	99.78	99.75	99.80	99.84
