# Supplementary material for: Efficiency and Power as a Function of Sequence Coverage, SNP Array Density, and Imputation
Source: PLoS Comput Biol. 2012 Jul 12;8(7):e1002604. doi: 10.1371/journal.pcbi.1002604 (PMC3395607; doi:10.1371/journal.pcbi.1002604)
Supplement: Figure S14 — Sensitivity and specificity by minor allele frequency: 381 European sample reference panel. Shown are data analogous to Figure 2c but with SpecI in addition to SensI. (PDF) [file pcbi.1002604.s014.pdf]

Sensitivity and specificity by minor allele frequency

381 European sample reference panel

$Sens_I$ for private variants

	0x	.5x	1x	2x	4x
No Array	NA	10.96	18.58	28.85	46.26
Affy 100k	0.46	11.06	18.69	30.53	45.18
Affy 500k	3.08	11.96	20.94	29.73	44.34
Affy 6	4.13	13.49	20.97	31.32	44.87
Ilmn 1M	4.07	13.69	21.10	29.42	47.58
Omni 2.5	5.57	16.21	22.83	31.97	47.20

$Spec_I$ for private variants

	0x	.5x	1x	2x	4x
No Array	NA	98.22	99.11	99.47	99.68
Affy 100k	100.00	98.25	99.13	99.50	99.67
Affy 500k	92.70	98.45	99.21	99.49	99.67
Affy 6	95.00	98.59	98.39	99.50	99.67
Ilmn 1M	95.00	98.67	99.20	99.47	99.35
Omni 2.5	96.48	98.91	99.27	99.52	99.68

$Sens_I$ for .5% $<$ MAF $<$ 5% variants

	0x	.5x	1x	2x	4x
No Array	NA	63.17	70.77	79.61	86.72
Affy 100k	17.94	64.27	70.29	79.21	86.19
Affy 500k	49.06	66.84	71.44	80.06	86.61
Affy 6	57.66	68.39	72.69	80.27	87.19
Ilmn 1M	62.71	70.26	73.70	80.37	86.65
Omni 2.5	69.20	73.77	76.17	82.27	87.65

$Spec_I$ for .5% $<$ MAF $<$ 5% variants

	0x	.5x	1x	2x	4x
No Array	NA	99.45	99.34	99.57	99.53
Affy 100k	98.75	99.35	99.42	99.51	99.48
Affy 500k	99.15	99.29	99.40	99.59	99.50
Affy 6	99.42	99.45	99.51	99.54	99.57
Ilmn 1M	99.48	99.49	99.47	99.52	99.54
Omni 2.5	99.51	99.63	99.57	99.67	99.64

$Sens_I$ for MAF $>$ 5% variants

	0x	.5x	1x	2x	4x
No Array	NA	87.47	92.49	95.22	96.96
Affy 100k	26.93	87.98	92.68	95.22	96.91
Affy 500k	74.08	90.85	93.77	95.44	96.98
Affy 6	86.55	92.82	94.51	95.91	97.17
Ilmn 1M	93.58	95.39	95.91	96.71	97.66
Omni 2.5	96.07	96.57	96.80	97.21	97.87

$Spec_I$ for MAF $>$ 5% variants

	0x	.5x	1x	2x	4x
No Array	NA	99.42	99.50	99.70	99.75
Affy 100k	97.47	99.34	99.50	99.68	99.73
Affy 500k	98.62	99.40	99.49	99.61	99.71
Affy 6	99.26	99.50	99.49	99.63	99.68
Ilmn 1M	99.67	99.73	99.75	99.78	99.81
Omni 2.5	99.77	99.79	99.78	99.81	99.85
