# Supplementary material for: Efficiency and Power as a Function of Sequence Coverage, SNP Array Density, and Imputation
Source: PLoS Comput Biol. 2012 Jul 12;8(7):e1002604. doi: 10.1371/journal.pcbi.1002604 (PMC3395607; doi:10.1371/journal.pcbi.1002604)

Sensitivity and specificity at sites not on the array: actual values

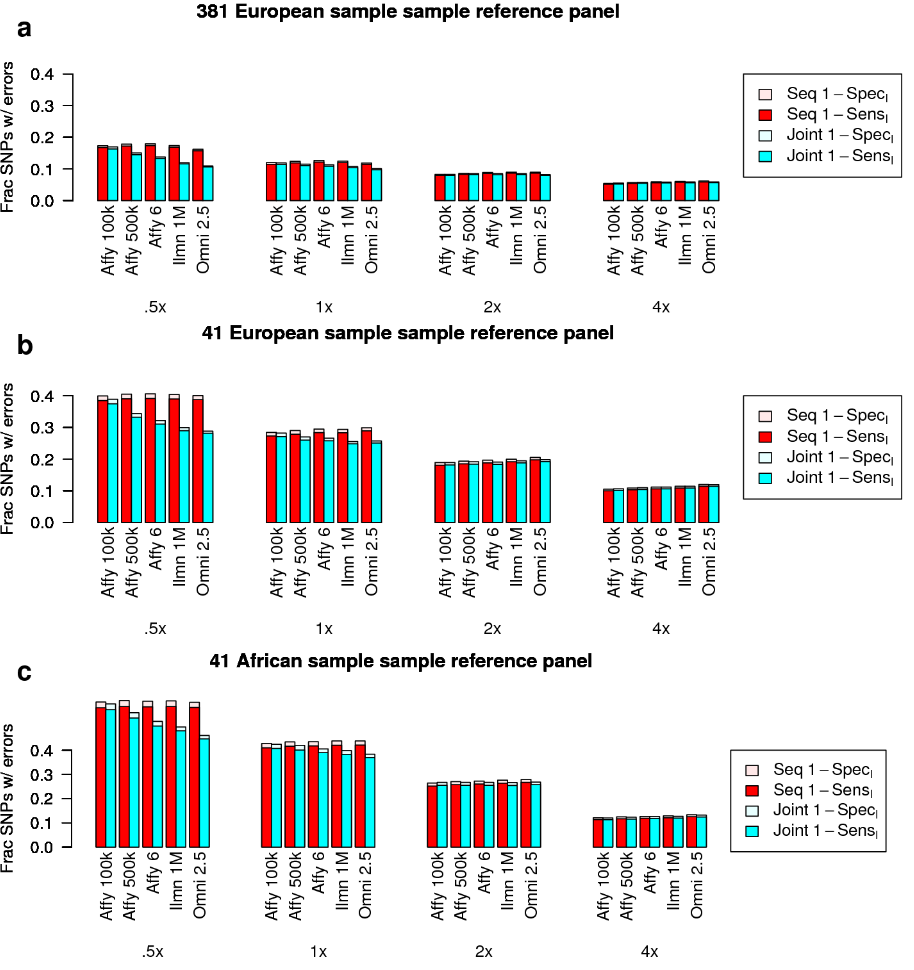

Supplement: Figure S16 — Sensitivity and specificity at sites not on the array: actual values. Shown is data analogous to Figure 4a but with actual values rather than normalized values. Calls based on sequence data (Seq) calls are plotted in red, joint (Joint) calls are plotted in blue. The red bars differ in size because the sites analyzed depend on the array. (a) 381 European sample reference panel. (b) 41 European sample reference panel. (c) 41 African sample reference panel. (PDF) [file pcbi.1002604.s016.pdf]
