# Supplementary material for: Efficiency and Power as a Function of Sequence Coverage, SNP Array Density, and Imputation
Source: PLoS Comput Biol. 2012 Jul 12;8(7):e1002604. doi: 10.1371/journal.pcbi.1002604 (PMC3395607; doi:10.1371/journal.pcbi.1002604)
Supplement: Figure S20 — Impact of new clusters and joint likelihoods on sensitivity and specificity at sites on the chip. At sites on the SNP array, joint calls have higher sensitivity and specificity than array calls for two reasons: genotype likelihoods for the test-sample are computed from both sequence and array data, and genotype cluster locations are computed based on sequence data and haplotype phasing (Figure 1, Figure S1). To quantify these two contributions, we compared sensitivity and specificity for array calls (Array only), for calls made from the joint likelihoods without cluster re-estimation (Likelihoods only), for calls made from array likelihoods but with cluster locations re-estimated from all data (Cluster only), and for joint calls (Joint). We did not use haplotype phasing to compute any genotype calls for this experiment. Joint calls were made in the same manner as described in Figure 4b. (PDF) [file pcbi.1002604.s020.pdf]

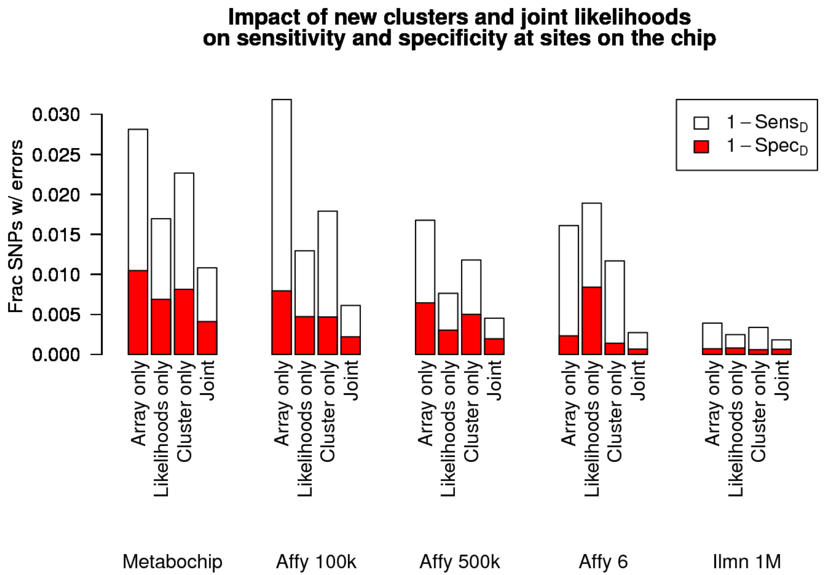
Impact of new clusters and joint likelihoods
on sensitivity and specificity at sites on the chip
Frac SNPs w/ errors
0.000
0.005
0.010
0.015
0.020
0.025
0.030
1 – Sens_D
1 – Spec_D
Array only
Likelihoods only
Cluster only
Joint
Metabochip
Affy 100k
Affy 500k
Affy 6
Ilmn 1M
