# Supplementary material for: Efficiency and Power as a Function of Sequence Coverage, SNP Array Density, and Imputation
Source: PLoS Comput Biol. 2012 Jul 12;8(7):e1002604. doi: 10.1371/journal.pcbi.1002604 (PMC3395607; doi:10.1371/journal.pcbi.1002604)

a Mathematics of fragment-based calling

$$\Pr\{f_i \mid GT_{AB}\} = \frac{\Pr\{f_i \mid A\}}{2} + \frac{\Pr\{f_i \mid B\}}{2}$$

where $$\Pr\{f \mid B\} = \sum_{i=A,C,G,T} \left\{ \Pr\{f_{i\_sequenced} \mid B_{true}\} * \prod_{j=b_1,b_2} \Pr\{b_j \mid f_i\} \right\}$$

$$\Pr\{f_{sequenced} \mid B_{true}\} = \left\{ \begin{array}{ll} 1-p & f \equiv B \\ p/3 & otherwise \end{array} \right\} \qquad \Pr\{b \mid f\} = \left\{ \begin{array}{ll} 1-e & b \equiv f \\ e/3 & otherwise \end{array} \right\}$$

b SNP genotype likelihood quality

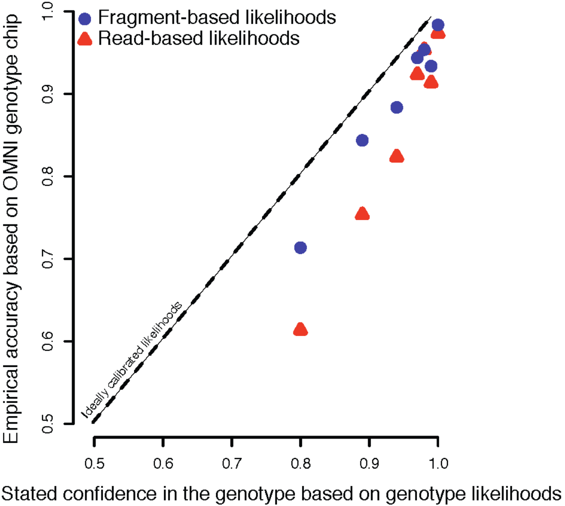

Supplement: Figure S22 — Genotype likelihoods of fragment-based calling. (a) Mathematical formalism for fragment-based (rather than read-based) SNP calling. The likelihood of a read pair given a hypothesized genotype GTAB with alleles A and B is calculated via a two-stage inference that weights the probability of each read independently by the probability of a PCR (or other) error occurring in the sequenced DNA fragment. In the above equation, p refers to error rate of the fragment, f refers to the base in the fragment, e refers to the error rate in the read, and b refers to the base in the read. Thus, errors that occur during fragment construction are counted only once, while errors that occur during sequencing are counted independently. (b) Comparison of the SNP genotype likelihood quality for GATK [27] SNP calls at sites also on the Omni 2.5 array (chromosome 20 only). Ideally calibrated likelihoods would follow the diagonal line. Fragment-based likelihoods are more accurate at all confidence levels, but the impact is most important for low confidence levels — which correspond to points with less certain likelihoods. (PDF) [file pcbi.1002604.s022.pdf]
