# Supplementary material for: Efficiency and Power as a Function of Sequence Coverage, SNP Array Density, and Imputation
Source: PLoS Comput Biol. 2012 Jul 12;8(7):e1002604. doi: 10.1371/journal.pcbi.1002604 (PMC3395607; doi:10.1371/journal.pcbi.1002604)
Supplement: Figure S23 — Analysis of disputed SNPs. As described in the text, we used our framework to resolve SNPs where array data widely disagreed with 4× sequencing data. We classify disputes as apparent sequence (or array) errors if the joint genotype calls disagree with calls based on sequence (or array) data. (a) Example disputed SNPs. The left plots show calls based on array data (ovals represent genotype classes) and sequence data (outlines of circles represent genotype classes); the right plots show joint calls with colors and symbols as defined in Figure S1. (bc) We asked which disputed SNPs were due to known or predicted error modes. Plotted are values for six potential error modes, each stratified across four classes of SNPs: agreed (sequence and array calls disagree for <10% of genotypes), disputed (sequence and array calls disagree for ≥10% of genotypes), apparent array errors, and apparent sequence errors. (b) Shown are the fraction of SNPs in each class (1) with a DNA flanking region that occurs at multiple genomic locations, (2) that lie within low complexity regions, and (3) that lie within 50 bp of another SNP. Apparent array errors have higher values for each of these metrics than apparent sequence errors. (c) Shown are distributions over SNPs for (1) the likelihood of forward/reverse DNA strand bias in sequence calls, (2) the length of a neighboring homopolymer run, and (3) the average (Phred-scaled) error rate of the reads supporting the sequence call (higher values signify more confident SNP calls). Apparent sequence errors have higher values for each of these metrics than apparent array errors. (PDF) [file pcbi.1002604.s023.pdf]

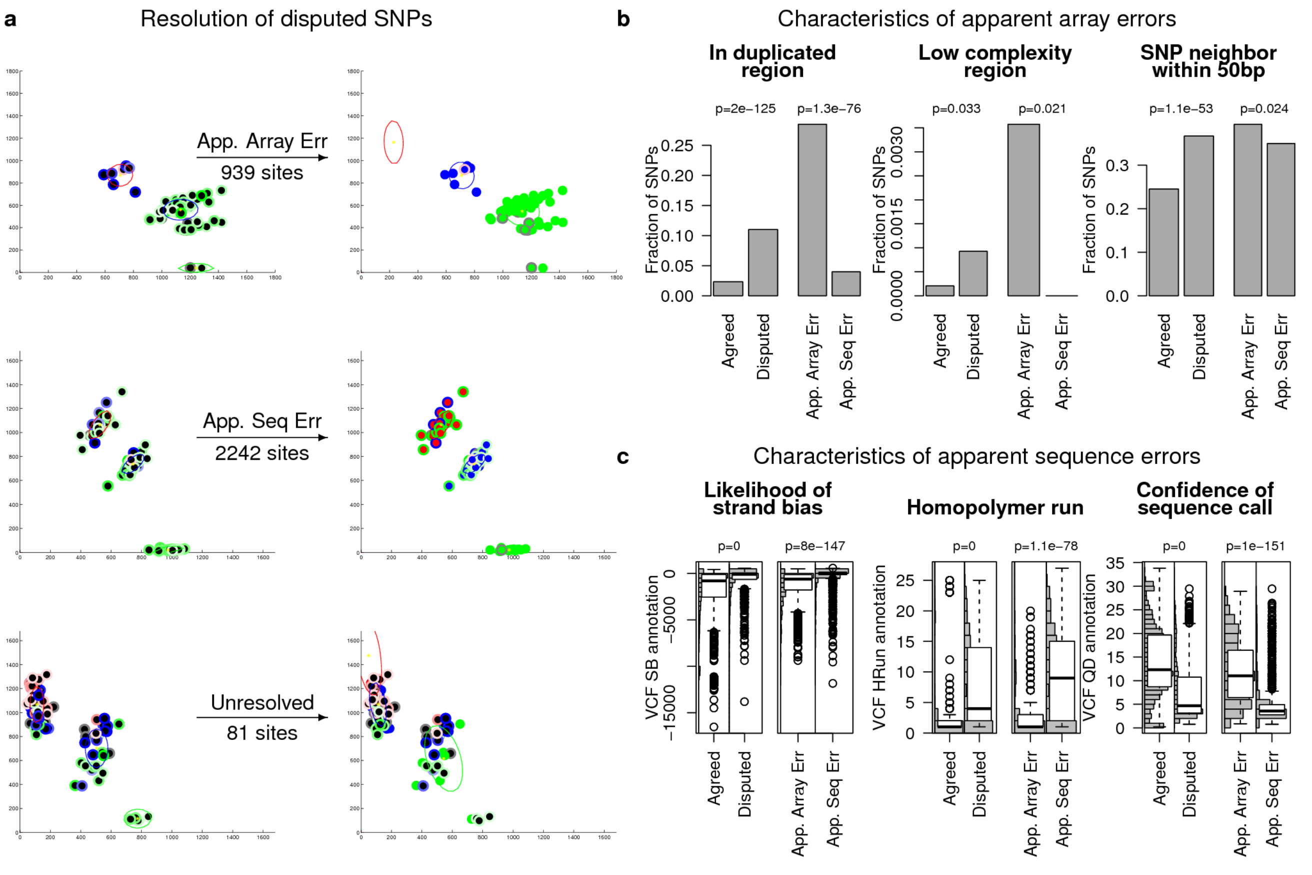

a
Resolution of disputed SNPs
App. Array Err
939 sites
App. Seq Err
2242 sites
Unresolved
81 sites
b
Characteristics of apparent array errors
In duplicated region
Low complexity region
SNP neighbor within 50bp
p=2e−125
p=1.3e−76
p=0.033
p=0.021
p=1.1e−53
p=0.024
Fraction of SNPs
Agreed
Disputed
App. Array Err
App. Seq Err
c
Characteristics of apparent sequence errors
Likelihood of strand bias
Homopolymer run
Confidence of sequence call
p=0
p=8e−147
p=0
p=1.1e−78
p=0
p=1e−151
VCF SB annotation
VCF HRun annotation
VCF QD annotation
