# Supplementary material for: Efficiency and Power as a Function of Sequence Coverage, SNP Array Density, and Imputation
Source: PLoS Comput Biol. 2012 Jul 12;8(7):e1002604. doi: 10.1371/journal.pcbi.1002604 (PMC3395607; doi:10.1371/journal.pcbi.1002604)
Supplement: Figure S26 — Characterized disputed SNPs. We fit separate distributions to each potential error mode for disputed and non-disputed SNPs, as well as for each class of apparent error mode. We considered a SNPs as characterized by an error mode according to one of two criteria: if the error mode was binary, it characterized a SNP if the SNP had value 1 for the error mode; if the error mode was continuous, it characterized a SNP if the log of the likelihood ratio (LOD) of the error mode distribution to the non-disputed distribution exceeded two. (a) SNPs with greater than 25% discordant genotypes. (b) SNPs with greater than 10% discordant genotypes. (PDF) [file pcbi.1002604.s026.pdf]

a

Number of characterized SNPs with ≥ 10% discordant genotypes (LOD 2)

Metric	Apparent Array Err (867 SNPs)	Apparent Seq Err (2245 SNPs)	Unresolved (204 SNPs)
In duplicated region	246	89	29
SNP neighbor within 50bp	340	784	89
SNP neighbor within 5bp	103	337	33
Mislabeled probe	8	118	24
Allele balance	0	191	4
Similar het/ref sequence base qualities	0	0	0
Homopolymer run	0	0	0
At most 2 haplotypes	2	38	24
Sequence read mapping quality	5	1	3
Sequence read mapping quality of zero	2	1	0
Similar het/ref read mapping qualities	0	0	0
Confidence of sequence call	0	0	0
Similar het/ref read position bias	3	117	15
Likelihood of strand bias	1	1	1
**Total**	**528**	**1114**	**143**

b

Number of characterized SNPs with ≥ 25% discordant genotypes (LOD 2)

Metric	Apparent Array Err (351 SNPs)	Apparent Seq Err (225 SNPs)	Unresolved (96 SNPs)
In duplicated region	142	13	10
SNP neighbor within 50bp	131	77	38
SNP neighbor within 5bp	33	25	18
Mislabeled probe	6	53	22
Allele balance	0	0	0
Similar het/ref sequence base qualities	0	0	0
Homopolymer run	0	0	0
At most 2 haplotypes	2	19	19
Sequence read mapping quality	3	0	1
Sequence read mapping quality of zero	1	1	0
Similar het/ref read mapping qualities	0	0	0
Confidence of sequence call	0	0	0
Similar het/ref read position bias	0	4	3
Likelihood of strand bias	0	6	0
**Total**	**241**	**148**	**72**
