# Supplementary material for: Efficiency and Power as a Function of Sequence Coverage, SNP Array Density, and Imputation
Source: PLoS Comput Biol. 2012 Jul 12;8(7):e1002604. doi: 10.1371/journal.pcbi.1002604 (PMC3395607; doi:10.1371/journal.pcbi.1002604)
Supplement: Text S1 — Available as supporting supporting information are: (1) calculations that show how previously described genotype calling algorithms are similar in principle to the joint calling framework when one or more of sequence reads, SNP array intensities, or linkage disequilibrium is omitted; (2) sensitivity and specificity calculations with a 41 sample European reference panel and a 41 sample African reference panel; and (3) investigation of the increase of sensitivity with additional genotyping investment. (PDF) [file pcbi.1002604.s027.pdf]

Text S1

Special cases of the joint calling framework

As described in Methods, given N samples and M SNPs, the joint calling framework computes $\Pr(G_n^m|\mathbf{I},\mathbf{S})$ — the posterior probability of the three genotypes for sample n at SNP m, conditional upon intensity data $\mathbf{I}$ and sequence data $\mathbf{S}$. This general computation uses sequence data, intensity data, and linkage disequilibrium (LD) to call genotypes. Omission of one or more of these data types produces special cases of the framework that are similar in principle to previously described algorithms.

Omission of sequence data and LD

Computation of $\Pr(G_n^m|\mathbf{I})$ without LD assumes that genotypes for different SNPs are independent. Therefore, SNP genotypes do not depend on intensity data from nearby SNPs and the calculation reduces to $\Pr(G_n^m|\mathbf{I}^m)$. SNP array genotyping algorithms, many of which have been described previously, compute this quantity. The Birdseed algorithm [1] uses an Expectation-Maximization (EM) algorithm closely analogous to that used by the full framework described in the Methods section.

Omission of intensity data and LD

Computation of $\Pr(G_n^m|\mathbf{S})$ without LD reduces to computation of $\Pr(G_n^m|S_n^m)$. Sequence genotype calling algorithms such as the Unified Genotyper in the Genome Analysis Toolkit [2], which we used to obtain sequence genotype likelihoods for our experiments, compute this quantity.

Omission of intensity data

Computation of $\Pr(G_n^m|\mathbf{S})$ with LD allows omission of the M-step from the full framework. The algorithm reduces to a single phasing and imputation step given the initial sequence likelihoods $\Pr(S_n^m|G_n^m)$. This previously proposed technique [3] was used to produce calls for the 1000 genomes project [4].

Omission of sequence data

Computation of $\Pr(G_n^m|\mathbf{I})$ with LD closely parallels the full computation of $\Pr(G_n^m|\mathbf{I},\mathbf{S})$ in the full framework. The modified E-step and M-step are:

E-step The E-step computes

$$Q(\theta^m|\theta^{m(t)}) = \mathrm{E}_{\mathbf{G}|\mathbf{I};\theta^{m(t)}}\left[\log L\left(\theta^m;\mathbf{I},\mathbf{G}\right)\right] \tag{1}$$

$$= \sum_n \sum_{i=1}^{3} \Pr\left(G_n^m = i|\mathbf{I};\theta^{m(t)}\right)\left[\log \pi_i^m + \log f\left(I_n^m;\mu_i^m,\Sigma_i^m\right)\right], \tag{2}$$

where $\Pr\left(\mathbf{G}|\mathbf{I};\theta^{m(t)}\right)$ is computed by a haplotype phasing and imputation algorithm.

M-step The parameters μ^m, Σ^m, and π^m are updated with

$$\theta^{m(t+1)} = \arg\max_{\theta^m} Q\left(\theta^m | \theta^{m(t)}\right),$$

which yields

$$\pi^{m(t+1)} = \frac{1}{N}\sum_{n=1}^{N} \Pr\left(G_n^m | \mathbf{I}; \theta^{m(t)}\right)$$

$$\mu_i^{m(t+1)} = \frac{\sum_{n=1}^{N} \Pr\left(G_n^m = i | \mathbf{I}; \theta^{m(t)}\right) I_n^m}{\sum_{n=1}^{N} \Pr\left(G_n^m = i | \mathbf{I}; \theta^{m(t)}\right)}$$

$$\Sigma_i^{m(t+1)} = \frac{\sum_{n=1}^{N} \Pr\left(G_n^m = i | \mathbf{I}; \theta^{m(t)}\right) \left(I_n^m - \mu_i^{m(t+1)}\right)\left(I_n^m - \mu_i^{m(t+1)}\right)^\top}{\sum_{n=1}^{N} \Pr\left(G_n^m = i | \mathbf{I}; \theta^{m(t)}\right)}$$

The BeagleCall algorithm performs a very similar computation, albeit with a different probabilistic model for intensity data [5].

Omission of LD

Computation of $\Pr\left(G_n^m | \mathbf{I}, \mathbf{S}\right)$ without reduces to computation of $\Pr\left(G_n^m | \mathbf{I}^m, \mathbf{S}^m\right)$. The modified E-step and M-step are:

E-step The E-step computes

$$Q(\theta^m | \theta^{m(t)}) = \mathrm{E}_{\mathbf{G}|\mathbf{I}^m, \mathbf{S}^m; \theta^{m(t)}} \left[\log L\left(\theta^m; \mathbf{I}^m, \mathbf{S}^m \mathbf{G}\right)\right] \tag{3}$$

$$= \sum_n \sum_{i=1}^{3} \Pr\left(G_n^m = i | \mathbf{I}^m, \mathbf{S}^m; \theta^{m(t)}\right) \left[\log \pi_i^m + \log f\left(I_n^m; \mu_i^m, \Sigma_i^m\right)\right], \tag{4}$$

where

$$\Pr\left(G_n^m | \mathbf{I}^m, \mathbf{S}^m; \theta^{m(t)}\right) = \Pr\left(G_n^m | I_n^m, S_n^m; \theta^{m(t)}\right) \tag{5}$$

$$\propto \Pr\left(G_n^m, I_n^m, S_n^m | \theta^{m(t)}\right) \tag{6}$$

$$= \Pr\left(I_n^m, S_n^m | G_n^m; \theta^{m(t)}\right) \Pr\left(G_n^m | \theta^{m(t)}\right) \tag{7}$$

$$= \Pr\left(I_n^m | G_n^m; \theta^{m(t)}\right) \Pr\left(S_n^m | G_n^m\right) \Pr\left(G_n^m | \theta^{m(t)}\right) \tag{8}$$

$$= f\left(I_n^m; \mu^{m(t)}, \Sigma^{m(t)}\right) \times \Pr\left(S_n^m | G_n^m\right) \times \pi^{m(t)}. \tag{9}$$

This computation is very similar to the E-step used by the Birdseed algorithm, but with the multiplicative factor $\Pr\left(S_n^m | G_n^m\right)$ used to further scale the genotype posterior probabilities.

M-step The parameters μ^m, Σ^m, and π^m are updated with

$$\theta^{m(t+1)} = \arg\max_{\theta^m} Q\left(\theta^m | \theta^{m(t)}\right),$$

which yields

$$\pi^{m(t+1)} = \frac{1}{N}\sum_{n=1}^{N} \Pr\left(G_n^m | \mathbf{I}^m, \mathbf{S}^m; \theta^{m(t)}\right)$$

$$\mu_i^{m(t+1)} = \frac{\sum_{n=1}^{N} \Pr\left(G_n^m = i | \mathbf{I}^m, \mathbf{S}^m; \theta^{m(t)}\right) I_n^m}{\sum_{n=1}^{N} \Pr\left(G_n^m = i | \mathbf{I}^m, \mathbf{S}^m; \theta^{m(t)}\right)}$$

$$\Sigma_i^{m(t+1)} = \frac{\sum_{n=1}^{N} \Pr\left(G_n^m = i | \mathbf{I}^m, \mathbf{S}^m; \theta^{m(t)}\right) \left(I_n^m - \mu_i^{m(t+1)}\right) \left(I_n^m - \mu_i^{m(t+1)}\right)^\top}{\sum_{n=1}^{N} \Pr\left(G_n^m = i | \mathbf{I}^m, \mathbf{S}^m; \theta^{m(t)}\right)}$$

To our knowledge, such an algorithm has not been previously described.

Impact of reference panel

For our main experiments, we varied the sequence and array data used for the test sample but used 4x sequence and 2.5m array data for the other 381 samples. This closely models the use of a public reference panel (from 1000G data) for imputation, although in practice the genotypes rather than the raw intensities and reads for the reference panel are input into a genotype calling framework.

Because for many studies a European reference panel may not be suitable, we used our framework to ask how results changed if an African reference panel was used for imputation.

Experimental data and procedure

We were not able to obtain data for 381 African samples to construct a reference panel of identical size to the one used in our main analysis. Therefore, we performed experiments with two additional reference panels, one African and one European, each with 41 unrelated samples from the Hapmap project [6]. Comparison between these two panels highlights differences that result from panel ethnicity, while comparison between the two European reference panels highlights differences that result from panel size.

Data for experiments with these two reference panels were obtained analogously to data for experiments with the 381 sample reference panel. The same intensity data was used or the smaller reference panels as for the larger reference panel. Sequence data was downloaded from the 1000G Pilot 1 release and down-sampled with the Genome Analysis Toolkit to approximate .5x, 1x, or 2x sequence coverage.

Two different test samples were used for the two different reference panels: Hapmap sample NA12878 for the European panel and Hapmap sample NA19240 for the African reference panel. Gold standard genotype and sequence data for these samples was downloaded from the 1000G Pilot 2 release. We down-sampled this data to approximate .5x (a random 1.875% of reads), 1x (3.75%), 2x (7.5%), or 4x (15%) sequence coverage for our test sample. This procedure was identical for these two test samples but slightly differed from the procedure used for the test sample in our main analysis; the European test sample had similar mean coverage across variant sites under the two different approaches. In addition, the African test sample had slightly higher coverage (1.3 times greater at variant sites) than the European test sample, which contributes to some of the differences between experiments that use the two different reference panels.

Results

We first find that, with a 41 European sample reference panel, Sens_I remains much higher than Sens_D but is significantly lower than Sens_I with a 381 European sample reference panel (Figure S4). The difference between the two European reference panels is significant even for the highest sequence coverage and SNP array density — for 4x sequence coverage and the 2.5m array, Sens_I decreases with reference panel size from 96.1% to 92.18%. However, the difference is larger for lower sequence coverage — for .5x sequence coverage it drops from 83.74% to 61.34% — or for SNP array data alone — for the 2.5m array it drops

from 91.93% to 78.55%. Spec_I is also lower with the smaller reference panel although values remain above 99% for most technologies.

Relative to the 41 European sample reference panel, the 41 African sample reference panel has lower Sens_I (Figure S5). Again, the difference is greatest for lower sequence coverages — the African reference panel yields Sens_I of 90.99% for 4x sequence data with the 2.5m array but only 41.15% for .5x sequence data or 61.43% for the 2.5m array alone. The African test sample has higher Sens_D than the European test sample as well as higher Sens_I for private variants, due in part to its slightly higher coverage. These results are consistent with previous observations that imputation with an African reference panel is less accurate than imputation with a European reference panel.

We also performed experiments with no reference panel. Here, we down-sampled (to .5x, 1x, or 2x) the entire set of 382 (or 42) samples and used phasing and imputation within the entire set. As expected, in this scenario we find an even more significant Sens_I drop-off relative to the 381 European reference panel (Figure S6). For sequence coverage below 4x, regardless of the SNP array used, Sens_I remains mostly well below 70%. These results show that the attraction of low-depth sequencing as a study design depends on a reference panel previously characterized with 4x sequence data.

As a final experiment, we explored the impact of different technologies used to build the reference panel (Figure S7). We find that the use of a 2.5m array and 4x sequence data to build the reference panel, rather than 4x sequence data alone, has a modest but positive impact on Sens_I and Spec_I. In general, our experiments show that reference panels built with different technologies change our quantitative results but keep our qualitative conclusions unchanged.

Return on investment

Our results show that, regardless of technology, additional data collection increases Sens_I. In general, additional data collection also increases a study's cost per sample. Therefore, studies face a trade-off between higher sensitivity and larger sample sizes.

We first asked at what point additional data collection yields only small gains in Sens_I. We used Sens_D as a measure of the amount of data collected, although it inexactly correlates with cost, because it is an intrinsic property of each technology. With a 381 European sample reference panel, we find that Sens_I quickly plateaus once Sens_D reaches about 50% (Figure S8). This number depends on the targeted frequency range: we observe a plateau for $> 5\%$ sites even for Sens_D values as low as 20%, while we do not observe any plateau for $< 5\%$ sites. These observations hold regardless of the specific combination of technologies used for data collection.

We also asked whether any data collection strategies of similar Sens_D have different Sens_I. We find that, for the most part, combinations with higher Sens_D have higher Sens_I. However, designs that use <500k arrays have lower Sens_I than designs with comparable Sens_D that do not use these arrays. Furthermore, for $< 5\%$ variants, designs with comparable Sens_D values but different density arrays have different Sens_I values — for example, 1x sequence data with a 1m SNP array has higher Sens_D but lower Sens_I than 2x sequence data alone.

References

1. Korn JM, Kuruvilla FG, McCarroll SA, Wysoker A, Nemesh J, et al. (2008) Integrated genotype calling and association analysis of SNPs, common copy number polymorphisms and rare CNVs. Nat Genet 40: 1253–1260.

2. DePristo MA, Banks E, Poplin R, Garimella KV, Maguire JR, et al. (2011) A framework for variation discovery and genotyping using next-generation DNA sequencing data. Nat Genet 43: 491–498.

3. Li Y, Sidore C, Kang HM, Boehnke M, Abecasis GR (2011) Low-coverage sequencing: implications for design of complex trait association studies. Genome Res 21: 940–951.

4. Altshuler D, Durbin RM, Abecasis GR, Bentley DR, Chakravarti A, et al. (2010) A map of human genome variation from population-scale sequencing. Nature 467: 1061–1073.

5. Browning BL, Yu Z (2009) Simultaneous genotype calling and haplotype phasing improves genotype accuracy and reduces false-positive associations for genome-wide association studies. Am J Hum Genet 85: 847–861.

6. Frazer KA, Ballinger DG, Cox DR, Hinds DA, Stuve LL, et al. (2007) A second generation human haplotype map of over 3.1 million SNPs. Nature 449: 851–861.
